# Dark-State-Mediated Low-Energy Electron Attachment to Molecular Oxygen: Symmetry Adapted Decomposition of the DEA Resonance through Dyson-Orbitals’ Incoherent Mixing

**DOI:** 10.1021/acs.jpclett.6c02057

**Published:** 2026-10-11

**Authors:** Narayan Kundu, Ayan Bhowmick, Aryya Ghosh, Dhananjay Nandi, Sourav Pal

**Affiliations:** † Department of Physics, 193131 Indian Institute of Science Education and Research, Kolkata 741246, India; ‡ 214794 University of Kassel, Institute of Physics, Heinrich-Plett-Str. 40, 34132 Kassel, Germany; ¶ Department of Chemistry, 448613 Ashoka University, Sonipat 131029, India

## Abstract

Dissociative electron attachment (DEA) to O_2_ exhibits a prominent resonance peak at ∼6.5 eV, producing O^–^ with a single kinetic energy band (∼1.5 eV) and a distinct four-lobed angular distribution. Using velocity-map imaging and complex absorbing potential equation-of-motion electron-attachment coupled-cluster singles and doubles (CAP-EOM-EA-CCSD) calculations, we notice that this resonance is mediated by electron-impact excitation to the optically dark *a* ^1^Δ_*g*_ and *b* ^1^Σ_*g*_^+^ states, which suggests electron impact excitation to these optically dark excited states provides an appropriate electronic structure for electron attachment forming ^2^Π_*u*_, ^2^Π_*g*_ and ^2,4^Σ_*u*_ Feshbach resonances. The measured angular distribution is reproduced by an incoherent superposition of three symmetry channels: ^2^Π_*u*_ (66.5%); ^2^Π_*g*_ (25.1%); and ^2,4^Σ_*u*_ (8.5%). The Dyson orbitals computed from the CAP-EOM-EA-CCSD calculations quantitatively reproduce the channel weights and their energy dependencies. This dark-state-mediated mechanism pinpointedly resolves the experimental observations and provides a general symmetry-adapted framework for decomposing DEA resonances from the fragmented anions angular distributions.

## Graphical abstract

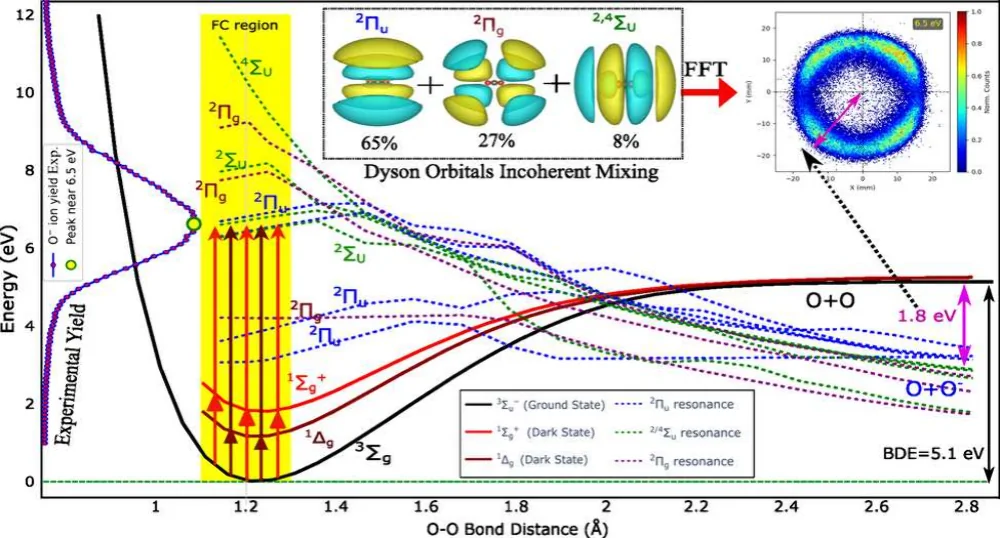

Dissociative electron attachment (DEA) captures a slow electron into a transient negative molecular anion that subsequently fragments:

1 $$O_{2}+e^{-}\to O_{2}^{-*}\to O^{-}+O$$

This same elementary step shapes negative-ion chemistry in the upper atmosphere,^1^ sets the electron balance of low-temperature plasmas,^2^ and drives DNA strand breaking induced by secondary electrons.^3–5^ Molecular oxygen has long served as the reference case: its resonance spectrum up to 15 eV has been catalogued by *R*-matrix theory,^6^ and its cross sections are tabulated to high precision.^1^

The resonance is unambiguously identified by three following experimental signatures. The O^–^ yield peaks sharply at 6.5 eV; the kinetic-energy release (KER) is a single narrow band at ∼1.5 eV (fwhm ≈ 0.3 eV), the hallmark of barrierless dissociation along a repulsive curve; and the angular distribution is distinctly four-lobed, with maxima near 45° and 135° and minima at 0°, 90° and 180° relative to the direction of the electron beam. These three observations cannot be reconciled with any single resonance. The symmetry of the resonance dictates the partial-wave expansion of the captured electron. For a homonuclear diatomic target, the *g*/*u* parity strictly selects the allowed angular momenta: a Σ_*g*_ state supports an *s*-wave (*l* = 0), leading to an isotropic distribution, while a Σ_*u*_ state is restricted to odd *l*, with the leading *p*-wave (*l* = 1, *m* = 0) producing a cos^2^θ pattern concentrated along the beam axis. In contrast, a pure Π state gives the familiar two-lobed sin^2^θ pattern.^7^ The early crossed-beam work of Van Brunt and Kieffer already showed that the O^–^ pattern from O_2_ demands more than one partial wave — a ^2^Π_*u*_ state built from *l* = 1 and *l* = 3, with the *l* = 3 weight growing toward higher energy^8^ — and *R*-matrix calculations later catalogued the relevant symmetry classes (^2^Π_*u*_, ^2^Π_*g*_, ^4^Σ_*u*_, ^2^Σ_*u*_).^6^ What has been missing is an account of how these symmetries combine in the observed yield and why the O^–^ cross section at 6.5 eV beam energy is so intense compared to other DEA resonances from diatomic molecules.

We trace the answer to a dark-state-mediated mechanism. The *a* ^1^Δ_*g*_ and *b* ^1^Σ_*g*_^+^ states of O_2_ are optically dark under electric-dipole selection rules; however, they are populated efficiently by electron impact. Unlike one-photon absorption, inelastic electron scattering is driven by the Coulomb interaction and is not restricted to the dipole approximation—exchange and higher-order multipole contributions provide efficient access to these metastable states.^9^ These states are extraordinarily long-lived, with radiative lifetimes running from seconds to minutes,^10^ making them highly effective reservoirs for subsequent attachment. Electron-impact excitation thus primes the molecule in a long-lived excited state, from which electron can attach to form a resonant transient negative anion on a repulsive potential curve. Routing the attachment through these excited states, rather than directly to the ground-state, enhances the effective DEA cross section well above what direct capture into the ground-state allows. Such intermediate-state gating is familiar from coherent-population-transfer schemes in atomic and molecular physics,^11^ and the controlling role of long-lived dark states in downstream dynamics is well established across photochemistry and radiation chemistry.^12^

The direct observation and characterization of optically dark states, however, are of fundamental importance in molecular dynamics as these electronic states, although not accessible by single-photon absorption, often determine important photochemical outcomes such as energy trapping, photostability, and overall reaction quantum yields.^13^ Recent work is important in that it goes beyond their elusive nature and resolves their dynamics and signatures experimentally. For example, studies have found dark states to be critical intermediates in light-driven molecular motors and have shown them to be the lowest-energy excited state in certain acenes.^14^ Dark states have been demonstrated to mediate photobleaching in fluorescent proteins and add an extra dimension to enhance imaging contrast.^15,16^ Thus, understanding the role of such dark states is not only a matter of fundamental curiosity but also a crucial step toward the rational design of more efficient molecular machines and optoelectronic materials.

The second contribution is of a methodological nature. In dissociative electron attachment, the observed O^–^ fragment can be supplied by several attachment pathways, each seeded by a different resonant precursor. In a single resonance, partial waves of the same symmetry interfere coherently, but partial waves of different symmetries have uncorrelated phase histories, and averaging over molecular orientation in the beam removes any cross-channel interference. Thus, the proper description is a two-level model: coherent within a channel, incoherent between channels — the exact structure of the O’Malley–Taylor symmetry-adapted angular-distribution theory.^7,17^ By contrast, photoelectron angular distributions (PADs) are described by a coherent sum over partial waves arising from a single initial state through dipole interaction.^18–20^ We use the experimental DEA data and apply the O’Malley–Taylor theory, fitting it using a SciPy global optimizer to extract the phase differences between partial waves for each resonance. The extracted momentum-space Dyson orbital weights from *ab initio* CAP-EOM-EA-CCSD calculations provide a practical route to dissect DEA in molecules too large for full potential-energy-surface mapping.^21–23^

All measurements were performed using a home-built velocity-map imaging (VMI) spectrometer in ultrahigh vacuum (base pressure ∼ 1 × 10^–9^ mbar). A pulsed electron beam (100 ns pulse width, 10 kHz repetition rate, energy spread 0.5–0.7 eV fwhm) crossed an effusive beam of pure O_2_ (99.999% purity, stagnation pressure 1.5 bar). Data acquisition was performed at a chamber pressure of ∼2 × 10^–7^ mbar to guarantee single-collision conditions. The details of the experimental setup were discussed in our past article.^24^

The O^–^ fragments from dissociative electron attachment were extracted by a two-stage ion lens and detected by a Z-stack microchannel plate (MCP) coupled to a hexanode delay-line anode from RoentDek. The detector measures the ion impact position (*x*-*y*) and the arrival time with a time-of-flight resolution better than 1 ns. Raw events were recorded in list-mode format using CoboldPC,^25^ which allows off-line full three-dimensional momentum reconstruction.

The electron energy scale was calibrated using the well-known O^–^/O_2_ DEA resonance at 6.5 eV.^26^ The data were recorded at three electron energies: 5.5, 6.5, and 7.5 eV. The radial ion distribution was translated into kinetic energy release (KER) and calibrated with known channels in DEA.^27^ Radial windows, narrow in width and centered on the KER peak, were integrated to obtain angular distributions *I*(θ), normalized to *I*(90°) = 1. For momentum imaging we used conical time-gated wedge-slice analysis^28–30^ and not conventional time-slice imaging. The latter is insensitive to the ionization volume and allows a quantitative comparison of the fragment yields at different momentum ranges at a specific electron energy. The design, calibration and operating parameters of the instrument have been discussed in our earlier publications.^24,28–30^

Figure 1 summarizes the experimental and theoretical picture. Panel (a) shows the measured ion-yield curve of O^–^/O_2_, peaking sharply near 6.5 eV as a DEA resonance. Panel (b) corresponds to the conical time-gated wedge-shaped velocity-slice image of O^–^. The four-lobed structure is evident at a glance: the O^–^ signal piles up along four lobes tilted 45° to the beam. All of the O^–^ signal falls on a single momentum ring, confirming a single kinetic-energy release. The theoretical potential energy curves calculated in panel (c) provide the mechanistic framework discussed below.

**Figure 1:**
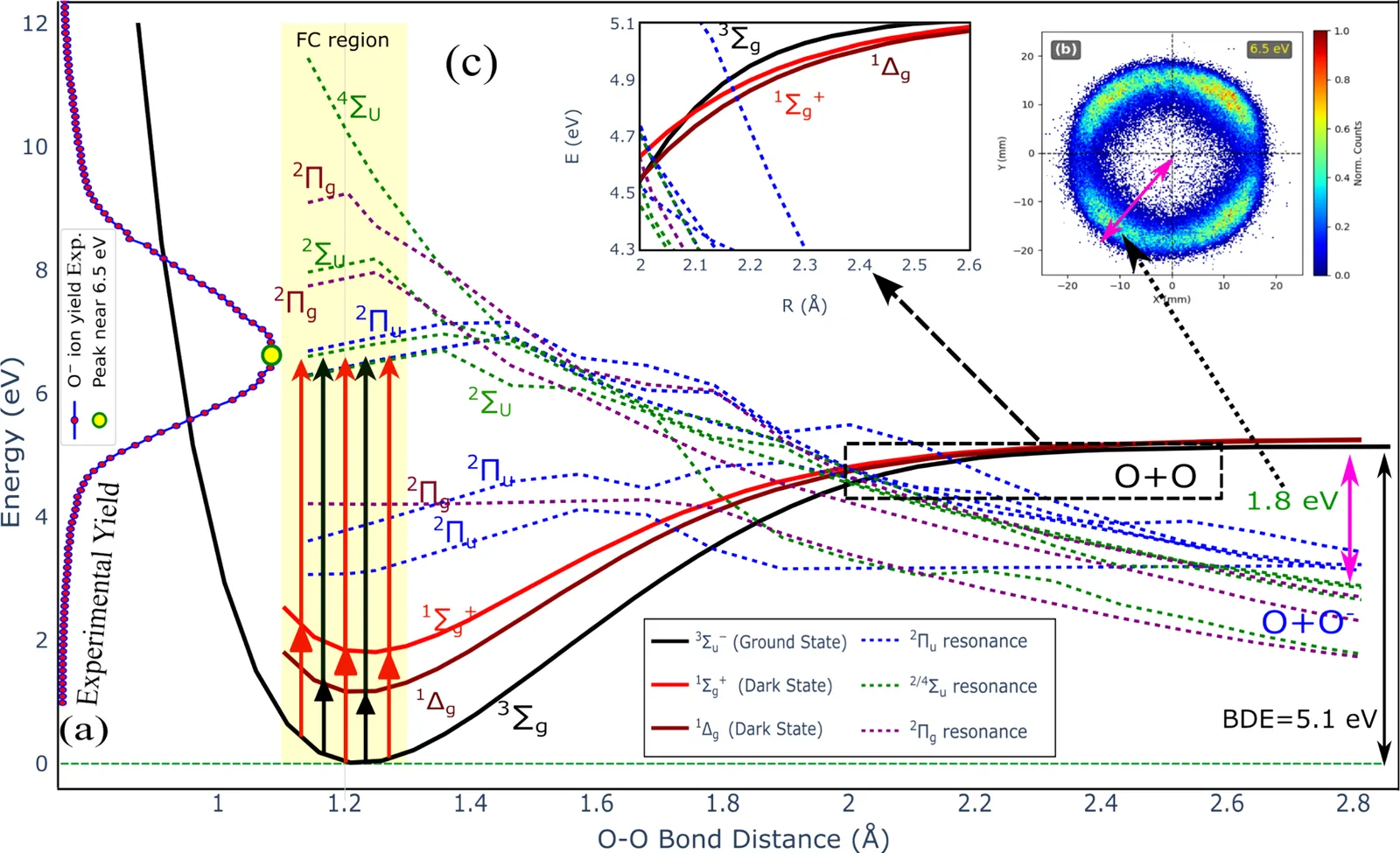
Experimental fingerprints and the dark-state picture for DEA to O_2_. (a) The measured O^–^ ion-yield curve, plotted along the energy axis, peaks near 6.5 eV (green marker); the shaded horizontal band marks the Franck–Condon (FC) region. (b) The 6.5 eV velocity-map image of the O^–^ fragment, with the magenta arrow along the lobe axis and the black arrow along the beam direction; the four-lobed pattern and the single momentum ring are evident. (c) Computed potential-energy curves: the solid curves are the CASPT2 neutral states — the *X* ^3^Σ_*g*_^–^ ground state (black) and the metastable *a* ^1^Δ_*g*_ (dark red) and *b* ^1^Σ_*g*_^+^ (red) dark states — and the dashed curves are the EOM-EA-CCSD anionic resonances, ^2^Π_*u*_ (blue), ^2^Π_*g*_ (magenta) and ^2/4^Σ_*u*_ (green). Vertical arrows indicate vertical electron attachment from the dark-state minima onto the repulsive ^2^Π_*u*_ wall; the magenta double arrow marks the 1.8 eV gap between the ^2^Π_*u*_ resonance and the O + O^–^ asymptote, and the bond dissociation energy is BDE = 5.1 eV. The inset zooms on the FC crossing region. Vertical attachment from the dark-state minima reaches the repulsive curve with no barrier, giving the observed single KER band of ∼1.5 eV.

For all three energies, the angular distributions, normalized to unity, are strongly four-lobed, with maxima at around 45°/135° and minima at 0°, 90° and 180°. Importantly, the intensity at 0°/180° is small but nonzero and increases monotonically from 5.5 to 7.5 eV due to increasing contribution from Σ_*u*_ coupling (μ = 0). This residual on-axis signal is a direct model-independent fingerprint of Σ_*u*_ coupling (μ = 0) and its growth follows the increasing Σ_*u*_ population at higher energy. Indeed, a pure Π distribution must vanish exactly along the beam axis.

The KER spectra at all energies display a single narrow band around 1.5 eV (fwhm ≈ 0.3 eV, instrument limited) without a low-energy tail. This means that the natural interpretation of this line shape is a single repulsive exit channel with no barrier, consistent with the dark-state picture developed below, which predicts such behavior.

To characterize the resonance states of O_2_^–^ and determine their resonance positions and decay widths, we employed the complex absorbing potential equation-of-motion electron-attachment coupled-cluster singles and doubles (CAP-EOM-EA-CCSD) method.^31–35^ Calculations were carried out using the aug-cc-pVTZ basis set augmented with an additional 3s3p3d diffuse basis placed on a ghost atom located at the midpoint of the O–O bond (O–O bond length = 1.205 Å). CAP calculations employed the projected CAP scheme proposed by Sommerfeld and Santra.^36^ A Voronoi-type complex absorbing potential (CAP) with a Voronoi cell radius of 5.0 Bohr was used to properly describe the metastable nature of the resonance states. The correspondence between the identified resonances and the underlying EOM-EA transitions is provided in the Supporting Information.

Within the CAP framework, resonance states are characterized by complex eigenvalues *E*(η) = *E*_*R*_ – *iΓ*/2, where *E*_*R*_ is the resonance position, Γ is the resonance width, and η denotes the CAP strength parameter. Since the complex energy depends on η, optimal CAP strengths (η*) are extracted from the stationary region of the CAP trajectory. CAP-EOM-EA-CCSD calculations were performed using Q-Chem 6.1, which provides uncorrected complex energies *E*(η) for a series of CAP strengths. The resulting η-trajectories for each electron-attached state were analyzed using the PyOpenCAP^37^ package. State tracking was carried out with the EigenvalueTrajectory module using the overlap-based tracking scheme, while first-order CAP corrections were applied using the derivative correction method.^38^ This yields the corrected complex energy *U*(η) = *E*(η) – *η dE*(η)/*dη*, which removes the leading-order η-dependent error introduced by the CAP. The optimal CAP strength (η*) was determined at the point satisfying min |*η dU*/*dη*|, corresponding to the stationary region of the corrected energy trajectory. The resonance position *E*_*R*_ and width Γ were then extracted from the corrected complex energy at this optimal CAP strength.

The nature of each resonance was determined from the excitation-amplitude composition of the EOM-EA-CCSD eigenvectors. Shape resonances were identified by dominant one-particle character, characterized by *R*_1_^2^ ≈ 1, whereas Feshbach resonances exhibit dominant two-particle–one-hole character, characterized by *R*_2_^2^ ≈ 1. The relative contributions of the *R*_1_ and *R*_2_ amplitudes were therefore used to distinguish between shape and Feshbach resonance states.

CAP-EOM-EA-CCSD calculations were performed on the ground electronic state of O_2_, *X* ^3^Σ_*g*_^–^. Since the reference state is a triplet, both α- and β-electron attachment channels were investigated. β-Electron attachment yields the ^2^Π_*u*_, ^2^Π_*g*_ and ^2^Σ_*u*_ resonance manifolds, whereas α attachment gives rise to the ^4^Σ_*u*_ resonances. All the CAP-EOM-EA-CCSD calculations along with Dyson orbitals were calculated at bond length of 1.205 Å.

In addition to the ground-state reference, CAP-EOM-EA-CCSD calculations were carried out on the excited *a* ^1^Δ_*g*_ and *b* ^1^Σ_*g*_^+^ state as the reference determinant. The *b* ^1^Σ_*g*_^+^ state was generated from the *a* ^1^Δ_*g*_ reference using Δ-SCF procedure and maximum-overlap constraints to prevent variational collapse, and this was achieved by enforcing the desired orbital occupation through molecular-orbital reordering.

The vertical excitation energies relative to the *X* ^3^Σ_*g*_^–^ ground state are 0.95 and 1.63 eV for the *a* ^1^Δ_*g*_ and *b* ^1^Σ_*g*_^+^ states, respectively, computed using XMS-CASPT2 ^39,40^ in a (12e,8o) active space, in good agreement with established spectroscopic values. For comparison, EOM-SF-CCSD calculations performed using the *X*,^3^Σ_*g*_^–^ ground state as the reference predict vertical excitation energies of 1.11 and 1.83 eV for the *a*,^1^Δ_*g*_ and *b*,^1^Σ_*g*_^+^ states, respectively. All calculations were performed within the *D*_2*h*_ Abelian subgroup of the full *D*_∞*h*_ molecular symmetry. The correlation between the irreducible representations of *D*_∞*h*_ and *D*_2*h*_, the corresponding EOM-EA transitions used to characterize each resonance, and details of the additional diffuse basis functions are provided in the Supporting Information.

Potential energy curves (PECs) of the neutral ground and excited electronic states of O_2_ were computed using the XMS-CASPT2 ^39,40^ method with a (12e,8o) active space and the aug-cc-pVTZ basis set, as implemented in OpenMOLCAS v26.02. The potential energy curves of the corresponding electron-attached states were calculated using the EOM-EA-CCSD method with the aug-cc-pVTZ basis set augmented by additional diffuse functions, as implemented in Q-Chem 6.1.

The resulting potential energy curves (Figure 1c) place the minima of the *a* ^1^Δ_*g*_ and *b* ^1^Σ_*g*_^+^ dark states near 1.21–1.22 Å, allowing vertical electron attachment at these geometries to access the ^2^Π_*u*_ resonance state, whose potential energy curve is entirely repulsive in the Franck–Condon region.

The angular distribution of O^–^ fragments from dissociative electron attachment to O_2_ is determined by the molecular-frame electron capture step under the axial-recoil approximation, valid because dissociation is fast compared to molecular rotation, so the O^–^ fragment recoils along the internuclear axis, taking the form *I*(θ) = ∑_μ=±Λ_|∑_*l*_ *a*_*l*,μ_ *e* ^*iδ*_*l*_^ *Y*_*l*,μ_(θ,0)|^2^.^7^ The projection Λ of the resonant electronic state angular momentum onto the molecular axis sets μ = Λ because the initial target state is ^3^Σ_*g*_^–^ (Ground State of O_2_), and the gerade/ungerade symmetry of the resonance restricts the sum to even *l* for *g* or odd *l* for *u* states, guaranteeing forward–backward symmetry. The partial-wave capture amplitudes *a*_*l*,μ_ are extracted from the Dyson orbital of the resonance by evaluating its three-dimensional Fourier transform on the momentum-space sphere of radius $k_{0}=\sqrt{2E_{\mathrm{inc}}}$ , after a phase correction that shifts the origin to the bond midpoint to ensure clean *g*/*u* separation. Full derivations are given in the SI.

Numerically, the Dyson orbital is zero-padded, transformed via FFT (Fast Fourier Transform), and spline-interpolated onto the *k*_0_-sphere; angular projection with spherical harmonics yields the amplitudes. For this calculation the Dyson orbitals *Re*(ϕ^*lr*^) were used from CAP-EOM-EA-CCSD calculation. Symmetry-forbidden (*l*, μ) channels are discarded, with a typical contamination below 1–2%. The magnitudes |*a*_*l*,μ_| are fully orbital-determined, while the scattering phase shifts δ_*l*_ are continuum quantities that must be supplied externally unless a single partial wave dominates, in which case the angular distribution is independent of δ_*l*_. When several resonance states contribute at overlapping energies, the total distribution is the incoherent, intensity-weighted sum

2 $$I_{\mathrm{total}}\left(\theta \right)=\underset{i}{\sum }w_{i}I_{i}\left(\theta \right)$$

where *w*_*i*_ reflects the relative cross section or branching ratio of each resonance channel *i*. The scattering phase shifts δ_*l*_ are taken from experimental data.

The CAP-EOM-EA-CCSD calculation on the ground state X^3^Σ_*g*_^–^ reproduces the known shape resonances ^2^Π_*g*_ at 0.43 eV (which is a bound state of O_2_^–^) and ^4^Σ_*u*_/^2^Σ_*u*_ around 1.5–2 eV as calculated by Ervin et al.^41^ (SI, Table S4). It also reproduces ^4^Σ_*u*_/^2^Σ_*u*_ in the 8–11 eV range as experimentally shown by Prabhudesai et al.^42^ The ^2^Π_*u*_ resonance obtained in the present calculations is identified as a Feshbach resonance, in agreement with the characterization proposed by Laporta et al.^6^ In the 5–8 eV window it predicts a ^2^Π_*u*_ resonance at 5.819 eV (Γ = 0.332 eV) and a second one at 7.538 eV (Γ = 0.890 eV). Hence, direct capture from the ground state gives rise to Feshbach resonances at 5.82 and 7.54 eV, but not at 6.5 eV. Therefore, direct attachment cannot explain the 6.5 eV band.^6^

We also calculated the electron attachments to the excited neutral states *a* ^1^Δ_*g*_ and *b* ^1^Σ_*g*_^+^ of oxygen. The resulting ^2^Π_*u*_ resonance is predicted to occur at **6.001** eV with Γ = 0.156 eV for attachment to the *a* ^1^Δ_*g*_ state, and at **6.270** eV with Γ = 0.028 eV for attachment to the *b* ^1^Σ_*g*_^+^ state. These resonance energies are in good agreement with the experimentally observed peak position near 6.5 eV beam energy and fall within the expected accuracy of the CAP-EOM-EA-CCSD methodology. Further investigation shows a Feshbach-type ^2^Π_*g*_ resonance at **6.241 eV** with Γ = 0.278 eV and a ^2^Σ_*u*_ resonance at **6.291 eV** with Γ = 0.736 eV for attachment to the *b* ^1^Σ_*g*_^+^ state. Complete-basis-set (CBS) extrapolations were performed for the ground and excited states of O_2_ (SI, Tables S8.1 and S8.1.1), as well as for the electron-attached (EA) states. For the EA states, only the real parts of the complex-absorbing-potential (CAP)-stabilized energies were considered for CBS extrapolation, as the imaginary parts, which are associated with the resonance widths, do not necessarily follow the same extrapolation scheme. Nevertheless, CBS-extrapolated energies of EA states should be interpreted with caution owing to their pronounced basis-set dependence. While CBS extrapolation generally provides reliable estimates for shape resonances, Feshbach resonances are particularly sensitive to both the basis set and the level of electronic-structure treatment. As a result, CBS-extrapolated Feshbach resonance energies may exhibit substantial quantitative deviations, even when the qualitative description remains reasonable.^43^ Furthermore, previous studies have shown that even higher-level treatments, such as EOM-CCSD­(T)­(a), can provide a qualitatively reasonable description of Feshbach resonances, while their predicted resonance energies and widths may not be quantitatively accurate.^43^ This dark-excited-state-mediated electron attachment provides a better electronic structure and elevates the scattering cross-section and resonance energy, which is reflected in our experimental observation. This fundamental mechanism was missing in our previous articles.^26,27^

Figure 2(a,b) compares the two- and three-channel fitting of the experimentally obtained 6.5 eV angular distribution of O^–^. The two-channel (Π_*u*_ + Π_*g*_) model fitting (panel a) is incomplete: it cannot reproduce the on-axis intensity and gives *R*_adj_^2^ = 0.5405. The addition of the Σ_*u*_ channel (panel b) changes the fit. The best model fit with 90° normalization yields

3 $$c_{\Pi_{u}}=66.5\%,c_{\Pi_{g}}=25.1\%,c_{\Sigma_{u}}=8.5\%$$

Here, (panel b) the model parameters: χ_red_^2^ = 0.983, weighted *R* ^2^ = 0.977 and adjusted *R* ^2^ = 0.974. The choice of the model is critical here, as the three-channel model reflects far better fit parameters compared to the best two-channel model. The three-channel model makes clear that the Σ_*u*_ channel is real and essential as a parsimonious description, well matched with our purely theoretically extracted angular distribution modeling in Figure 3 extracted from the same three-channel Dyson-orbital incoherent mixing, which reproduces the measured lobe positions and depths and their evolution with energy. The Σ_*u*_ channel contribution increases with beam energy. This is what the calculations anticipate, since increasing electron beam energy opens access to higher-lying ^4^Σ_*u*_^–^ and ^2^Σ_*u*_^–^ Feshbach resonances (Supporting Information, Table S4, at 11.075 and 9.801 eV).

**Figure 2:**
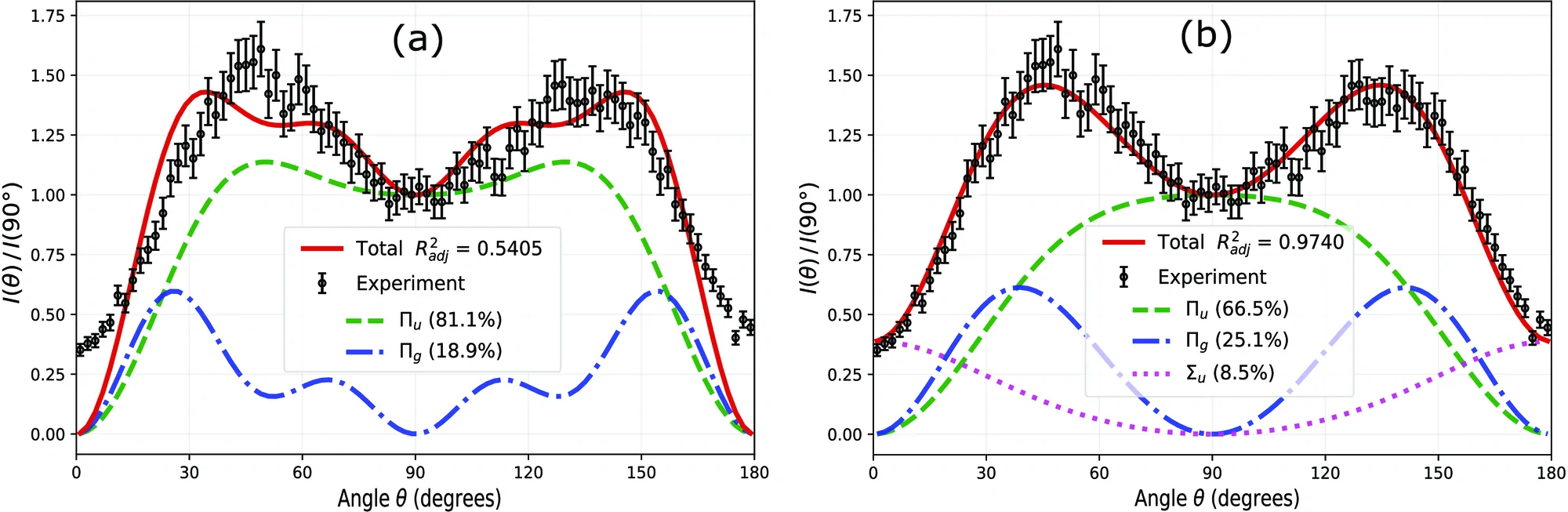
Symmetry decomposition of the O^–^ angular distribution from DEA to O_2_. (a) The experimental distribution at 6.5 eV (black circles, normalized to *I*(90°) = 1) fitted with only two channels, ^2^Π_*u*_ (green dashed, 81.1%) and ^2^Π_*g*_ (blue dash-dot, 18.9%); the total fit (red) is inadequate (*R*_adj_^2^ = 0.5405). (b) The same 6.5 eV data fitted with three incoherent channels, ^2^Π_*u*_ (66.5%), ^2^Π_*g*_ (25.1%) and Σ_*u*_ (magenta dotted, 8.5%); the total fit (red) is excellent (*R*_adj_^2^ = 0.9740).

**Figure 3:**
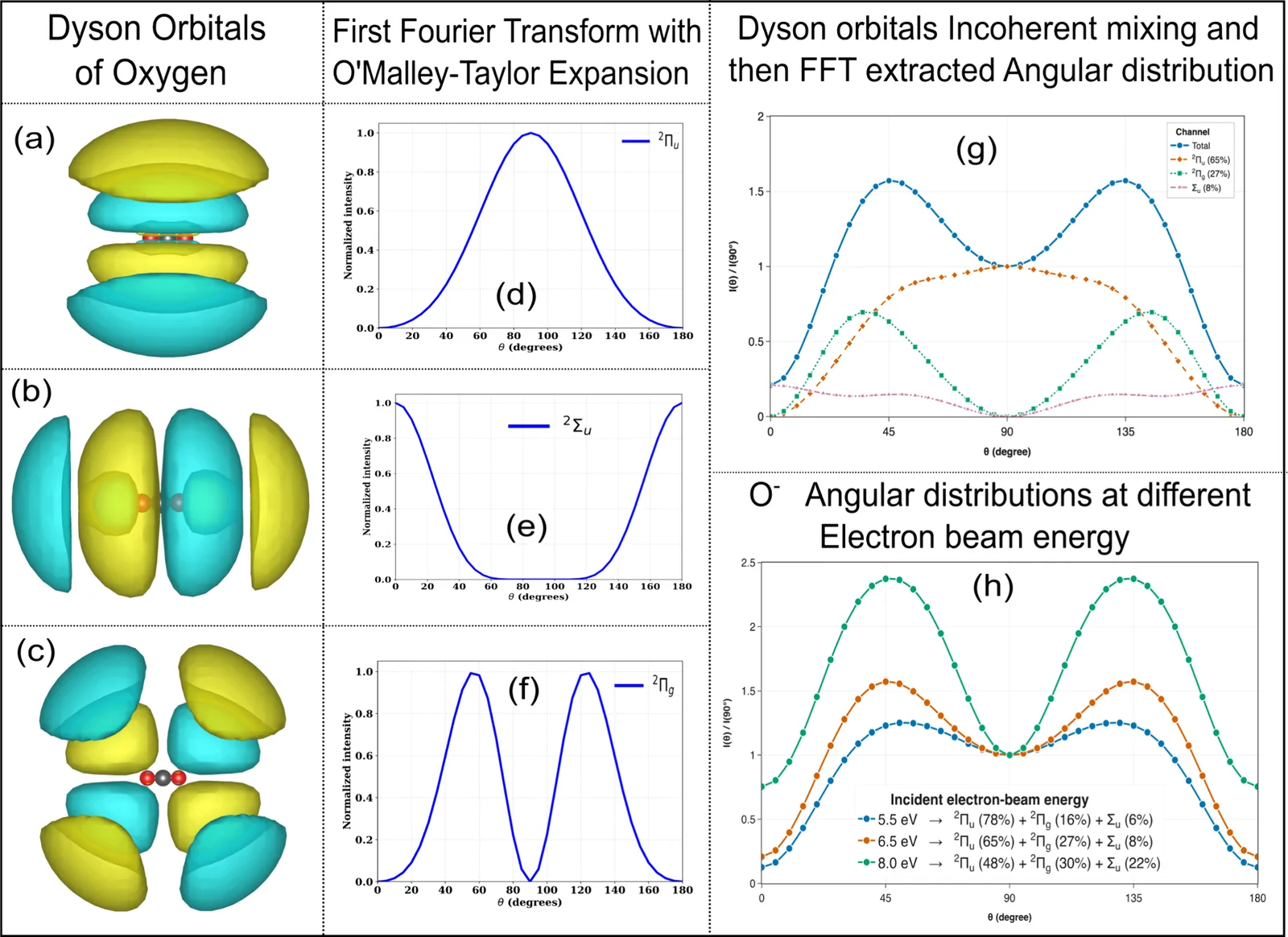
Eight-panel figure analyzing the Dyson decomposition and momentum-space angular distributions of O^–^. Panels (a), (b), and (c) show the angular distributions of individual Dyson orbitals from distinct ionization channels. Panels (d), (e), and (f) employ the same combined technique: first, a Fast Fourier Transform (FFT) of each Dyson orbital into momentum space, and second, the O’Malley-Taylor expansion of the resulting momentum-space angular distribution. Panel (g) reflects the angular distribution of O^–^ extracted from the three-channel Dyson-orbital incoherent mixing model. (h) Theoretical reconstructions built from the *ab initio* Dyson orbitals mixing at 5.5, 6.5, and 8.0 eV, with the symmetry weights indicated; the Σ_*u*_ contribution grows from 6% to 8% to 22% as the incident energy increases, matching well with the experimental observations.^26^

The fundamental difference between photoionization and dissociative electron attachment (DEA) angular distributions lies in their coherence structure. A photoelectron angular distribution (PAD) is a fully coherent sum over partial waves:

4 $$I_{\mathrm{PAD}}\left(\theta \right)={\left|\underset{l,m}{\sum }A_{lm}Y_{l}^{m}\left(\theta \right)\right|}^{2}$$

In DEA, the O’Malley–Taylor formalism describes the angular distribution as an incoherent sum over independent symmetry channels (e.g., ^2^Σ, ^2^Π), each channel labeled by a magnetic quantum number *m*_λ_ (*m* = 0 for Σ, |*m*| = 1 for Π) and containing a coherent sum over partial waves:

5 $$I_{\mathrm{DEA}}\left(\theta \right)=\underset{\lambda }{\sum }w_{\lambda }{\left|\underset{l}{\sum }c_{l,\lambda }Y_{l}^{m_{\lambda }}\left(\theta \right)\right|}^{2}$$

Cross-channel interference vanishes because different resonant precursors carry uncorrelated phase histories, and averaging over molecular orientation erases any remaining coherence between distinct symmetry channels.

Thus, by combining state-of-the-art high-resolution velocity-map imaging (VMI) with CAP-EOM-EA-CCSD calculations and an incoherent, symmetry-adapted Dyson-orbital mixing model, we have solved the long-standing puzzle of the 6.5 eV DEA resonance peak in O_2_. This resonance peak is mediated by optically dark states, where electron impact first populates the *a* ^1^Δ_*g*_ and *b* ^1^Σ_*g*_^+^ low-lying dark states, manifesting a better electronic structure for the electron capture into ^2^Π_*u*_ and ^2^Π_*g*_ Feshbach resonances at 6.001 and 6.241 eV respectively. An incoherent sum of ^2^Π_*u*_ (66.5%), ^2^Π_*g*_ (25.1%) and Σ_*u*_ (8.5%) reproduces the measured angular distribution at 6.5 eV (adjusted *R* ^2^ = 0.974, χ_red_^2^ = 0.983). The Σ_*u*_ weight increases with energy as higher-lying Σ Feshbach resonances open. The single ∼1.5 eV KER band arises from the repulsive anionic curve and the Franck–Condon window supplied by the dark state. This work goes beyond O_2_, providing a transferable recipe — the O’Malley–Taylor symmetry-adapted partial-wave expansion for Dyson orbitals, trained on the data with a SciPy optimizer — for reading the electronic-symmetry composition of a DEA resonance directly from its angular distribution, even when the full potential-energy surface is not accessible. This should help relate electron-driven chemistry, Quantum sensing mediated by coherent population trapping, from radiation damage in DNA to plasma processing of organic films, protein-folded health sciences to underlying molecular symmetries.

## Supplementary Material



## Notes

Optimized CAP-η trajectories; the *D*_∞*h*_ → *D*_2*h*_ symmetry-mapping table; the diffuse basis set; full resonance catalogs for *X* ^3^Σ_*g*_^–^, *a* ^1^Δ_*g*_ and *b* ^1^Σ_*g*_^+^ (positions, widths and shape/Feshbach assignments) (PDF)

## Abbreviations Used

DEA: dissociative electron attachment
VMI: velocity-map imaging
KER: kinetic energy release
CAP: complex absorbing potential
EOM-EA-CCSD: equation-of-motion electron-attachment coupled-cluster singles and doubles
ARA: axial recoil approximation
AIC: Akaike information criterion
BIC: Bayesian information criterion
PAD: photoelectron angular distribution
