# Supplementary material for: Dark-State-Mediated Low-Energy Electron Attachment to Molecular Oxygen: Symmetry Adapted Decomposition of the DEA Resonance through Dyson-Orbitals’ Incoherent Mixing
Source: J Phys Chem Lett. 2026 Oct 11;17(42):11998–2004. doi: 10.1021/acs.jpclett.6c02057 (PMC13700095; doi:10.1021/acs.jpclett.6c02057)
Supplement: Supplementary file 1 [file jz6c02057_si_001.pdf]

Supporting Information for

Dark-State-Mediated Low-Energy Electron Attachment to Molecular Oxygen: Symmetry Adapted Decomposition of the DEA Resonance through Dyson-Orbitals' Incoherent Mixing

Narayan Kundu* [1,2] Ayan Bhowmick [3], Aryya Ghosh,[3] Dhananjay Nandi[1] and Sourav Pal* [3]

[1]Department of Physics, Indian Institute of Science Education and Research, Kolkata 741246, India

[2]University of Kassel, Institute of Physics, Heinrich-Plett-Str. 40, 34132 Kassel, Germany

[3]Department of Chemistry, Ashoka University, Sonipat 131029, India

Section S1. Electronic Configuration

Oxygen ground state is $^3\Sigma_g^-([\mathrm{core}]1\pi_u^4 1\pi_g^2)$

Table S1.1. Electronic configurations of O2 resonances.

Resonance	Electronic Configuration
$^2\Pi_u$	$([\mathrm{core}]1\pi_u^3 1\pi_g^4)$
$^2\Pi_g$	$([\mathrm{core}]1\pi_u^4 1\pi_g^3)$
$^4\Sigma_u^-$	$([\mathrm{core}]1\pi_u^4 1\pi_g^2 3\sigma_u\uparrow)$
$^2\Sigma_u^-$	$([\mathrm{core}]1\pi_u^4 1\pi_g^2 3\sigma_u\downarrow)$

Section S2. Point Group

Symmetry mapping from $D_{\infty h}$ to D_{2h}

Table S2.1. Symmetry mapping from $D_{\infty h}$ to D_{2h} point group.

$D_{\infty h}$	D_{2h}
$^3\Sigma_g^-$	B_{1g}
$^2\Pi_u$	$B_{2u} + B_{3u}$
$^2\Pi_g$	$B_{2g} + B_{3g}$
$^4\Sigma_u^-$	A_u
$^2\Sigma_u^-$	A_u

Section S3. Diffused Basis Set on Ghost Atom

```
****
Gh   0
S    1    1.000000
2.88000000E-02    1.00000000E+00
S    1    1.000000
1.44000000E-02    1.00000000E+00
S    1    1.000000
0.72000000E-02    1.00000000E+00
P    1    1.000000
2.45000000E-02    1.00000000E+00
P    1    1.000000
1.22000000E-02    1.00000000E+00
P    1    1.000000
0.61000000E-02    1.00000000E+00
D    1    1.000000
0.755000000E-01   1.00000000E+00
D    1    1.000000
0.377500000E-01   1.00000000E+00
D    1    1.000000
0.188750000E-01   1.00000000E+00
****
$end
```

Section S4. Resonances from Ground State ($X^3\Sigma_g^- = B_{1g}$)

Ground state O_2 is a open shell system addition of β electron give rise to doublet resonances ($^2\Pi_u$, $^2\Pi_g$, $^2\Sigma_u^-$) and α electron to quartet resonances ($^4\Sigma_u^-$)

Table S4.1. EOMEA resonances from the ground state ($X^3\Sigma g^- = B1g$)

Resonances ($D_{\infty h}$)	D_{2h}	EOMEA Transition	State No.	Resonance Position (E_r) {eV}	Γ {eV}	Type
$^2\Pi_u$	$B_{2u} + B_{3u}$	B_{3u} *and* B_{2u}	3	1.880	0.585	Shape
			10	5.819	0.332	Feshbach
			12	7.538	0.890	Shape
			15	7.382	0.944	Feshbach
$^2\Pi_g$	$B_{2g} + B_{3g}$	B_{3g} *and* B_{2g}	1	0.430	0.011	Shape
			3	3.169	0.939	Feshbach
			7	7.017	0.680	Feshbach
			9	8.602	0.963	Feshbach
$^4\Sigma_u^-$	A_u	B_{1u}	3	1.641	0.728	Shape
			4	5.676	0.727	Shape
			9	11.075	0.132	Shape
$^2\Sigma_u^-$	A_u	B_{1u}	3	1.683	0.747	Shape
			7	5.842	0.711	Shape
			9	5.815	1.455	Feshbach
			12	9.801	0.729	Feshbach

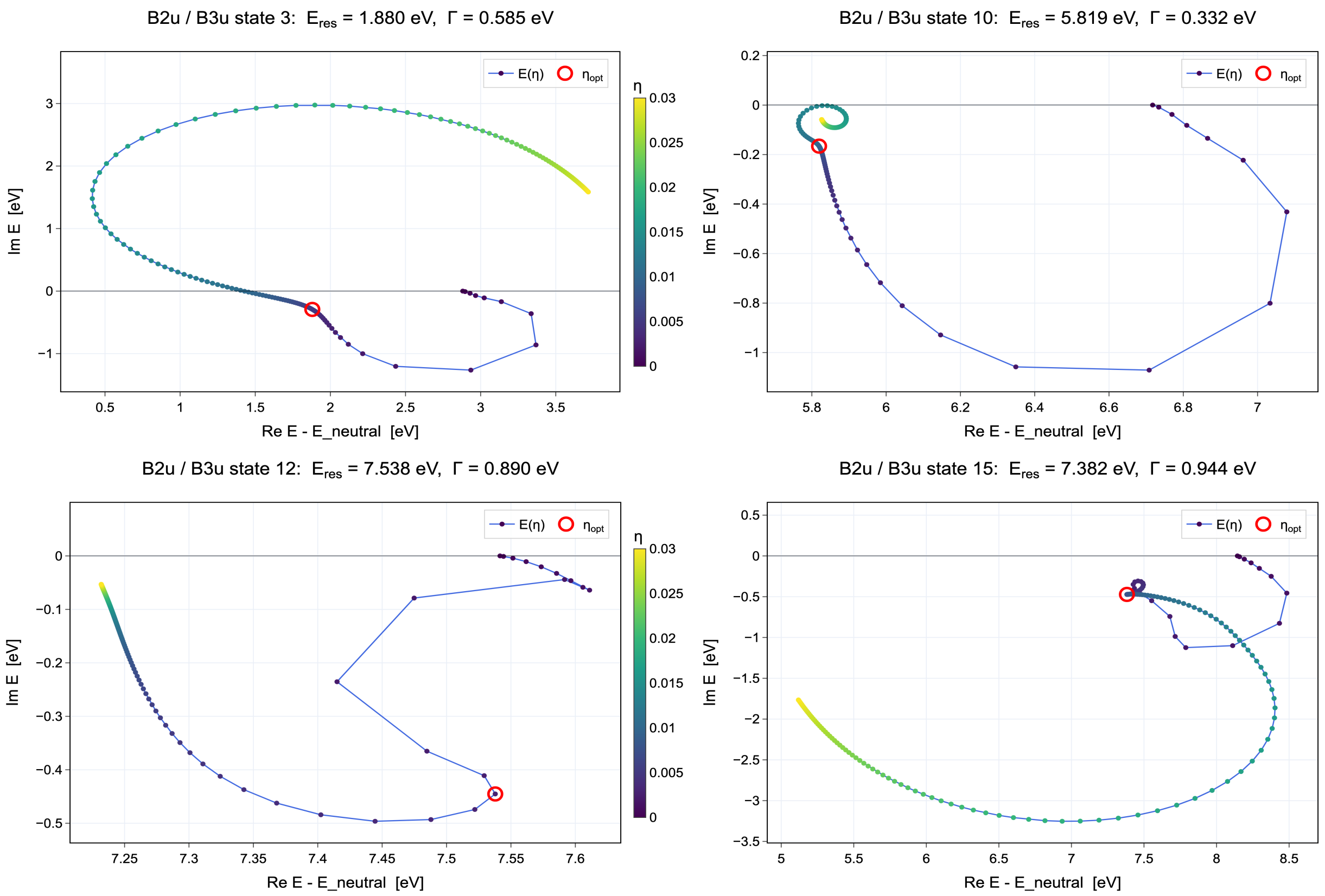

Fig S4.1. CAP η-trajectories for the degenerate B_{2u} and B_{3u} EOM-EA transitions obtained by β-electron attachment to the ground-state of O_2 , $X^3\Sigma_g^-$ The x-axis represents the real part of the resonance energy ($Re\ E$) relative to the neutral ground state, while the y-axis shows the imaginary part ($Im\ E$) The red circle denotes the optimal CAP strength (η_{opt}) determined from the trajectory analysis. The resonance energy (E_{res}) and decay width (Γ) evaluated at (η_{opt}) are indicated in the title of each panel.

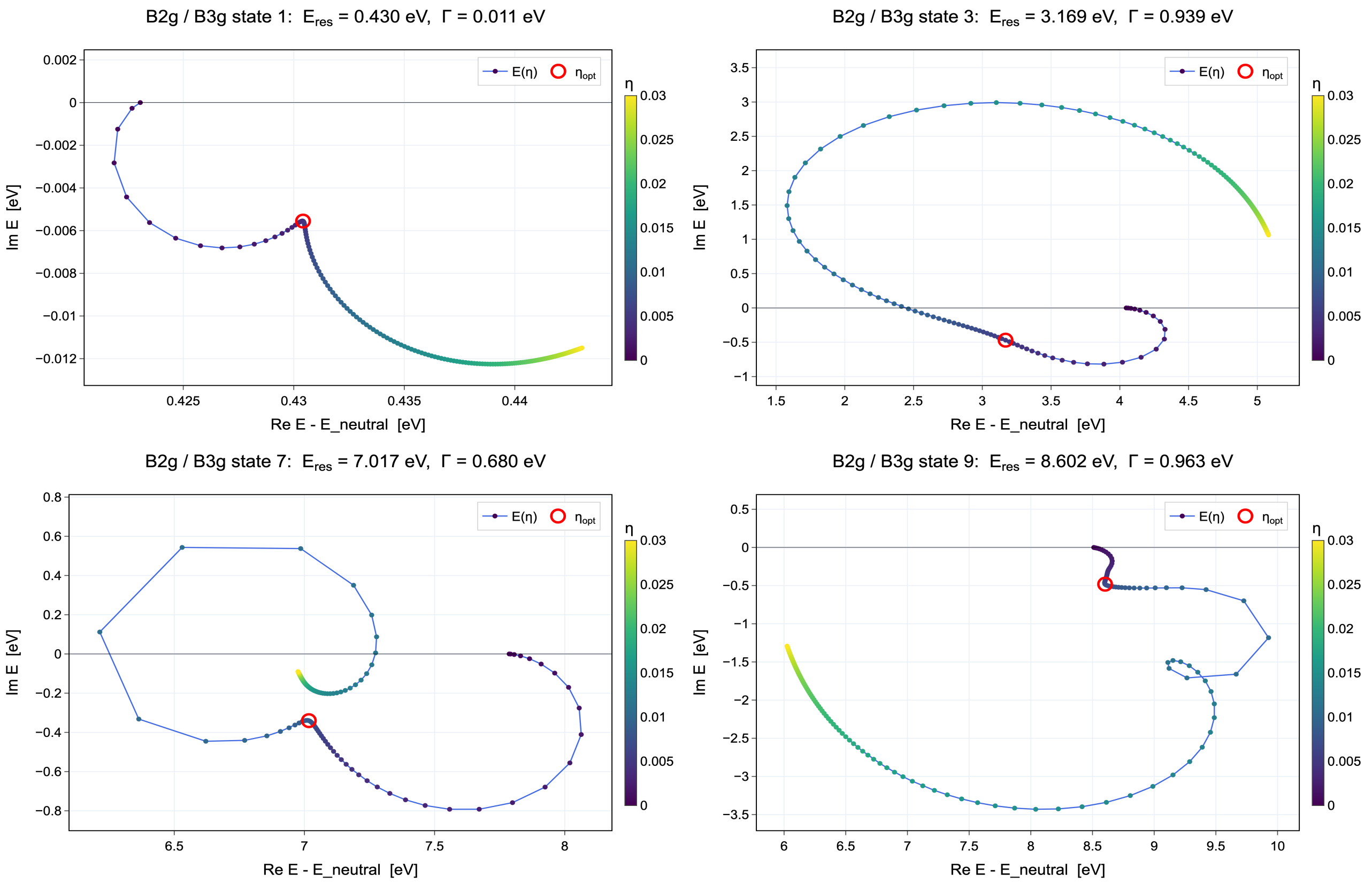

Fig S4.2. CAP η-trajectories for the degenerate B_{2g} and B_{3g} EOM-EA transitions obtained by β-electron attachment to the ground-state of O_2 , $X^3\Sigma_g^-$ The x-axis represents the real part of the resonance energy ($Re\ E$) relative to the neutral ground state, while the y-axis shows the imaginary part ($Im\ E$) The red circle denotes the optimal CAP strength (η_{opt}) determined from the trajectory analysis. The resonance energy (E_{res}) and decay width (Γ) evaluated at (η_{opt}) are indicated in the title of each panel.

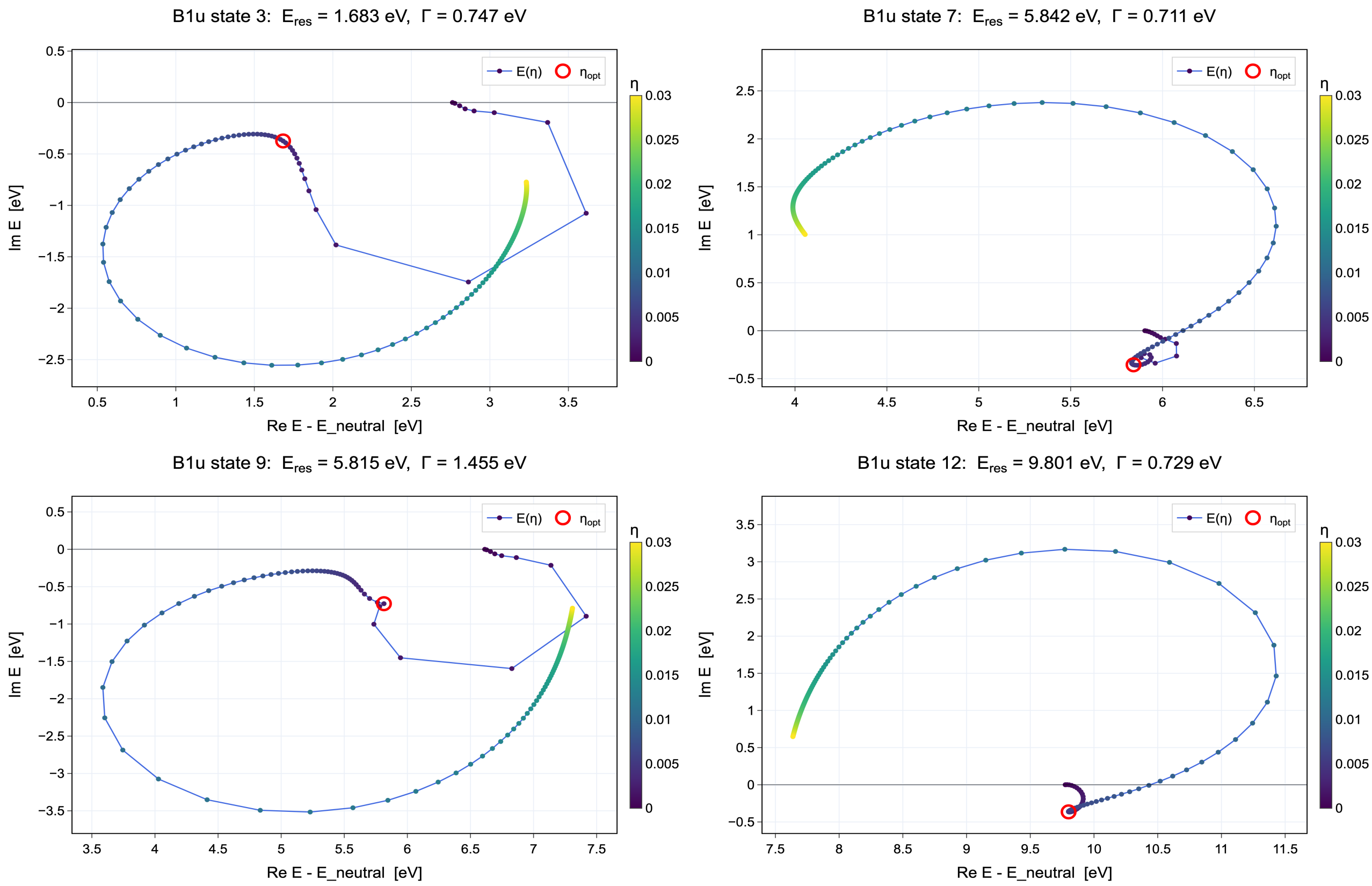

Fig S4.3. CAP η-trajectories for the B_{1u} EOM-EA transitions obtained by β-electron attachment to the ground-state of O_2 , $X^3\Sigma_g^-$ The x-axis represents the real part of the resonance energy ($Re\ E$) relative to the neutral ground state, while the y-axis shows the imaginary part ($Im\ E$) The red circle denotes the optimal CAP strength (η_{opt}) determined from the trajectory analysis. The resonance energy (E_{res}) and decay width (Γ) evaluated at (η_{opt}) are indicated in the title of each panel.

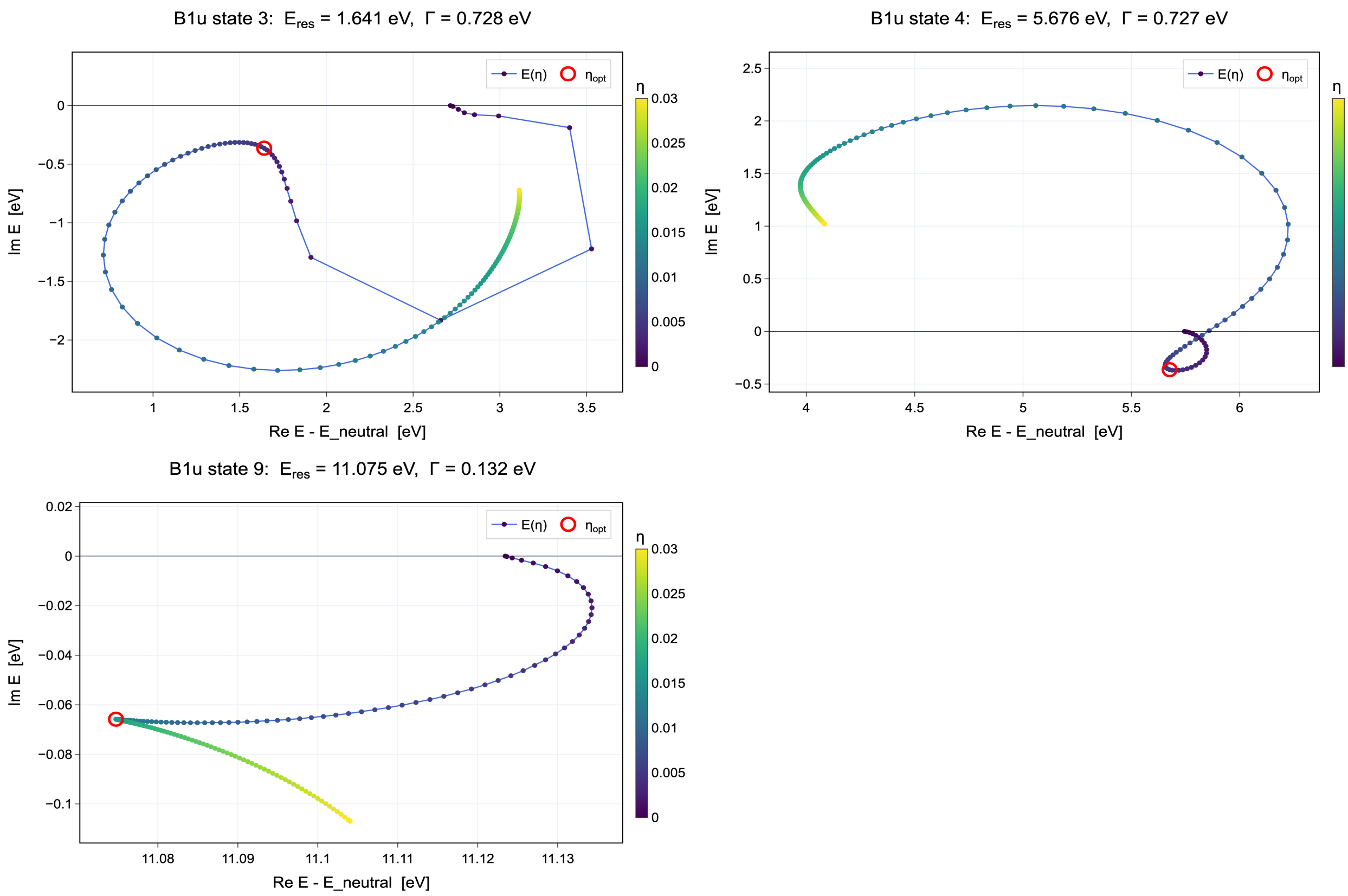

Fig S4.4. CAP η-trajectories for the B_{1u} EOM-EA transitions obtained by α-electron attachment to the ground-state of O_2 , $X^3\Sigma_g^-$ The x-axis represents the real part of the resonance energy ($Re\ E$) relative to the neutral ground state, while the y-axis shows the imaginary part ($Im\ E$) The red circle denotes the optimal CAP strength (η_{opt}) determined from the trajectory analysis. The resonance energy (E_{res}) and decay width (Γ) evaluated at (η_{opt}) are indicated in the title of each panel.

Section S5. Resonances from Excited State ($a^1\Delta_g = Ag + B1g$)

α and β electron attachment are equivalent

Table S5.1. EOMEA resonances from the Excited State ($a^1\Delta_g$)

Resonances ($D_{\infty h}$)	D_{2h}	EOMEA Transition	State No.	Resonance Position (E_r) {eV}	Γ {eV}	Type
$^2\Pi_u$	$B_{2u} + B_{3u}$	B_{2u}	5	3.334	0.588	Shape
			**11**	**6.001**	**0.156**	**Feshbach**
			12	7.470	0.328	Feshbach
			13	8.848	0.973	Shape
		B_{3u}	3	3.233	0.558	Shape
			11	5.971	0.276	Feshbach
			12	7.451	0.373	Feshbach
			13	8.529	0.954	Shape
$^2\Pi_g$	$B_{2g} + B_{3g}$	B_{2g}	1	0.515	0.003	Shape
			5	4.531	0.961	Shape
			8	7.185	0.608	Feshbach
			11	8.634	0.187	Feshbach
			12	9.889	1.009	Shape
			15	10.809	0.213	Feshbach
		B_{3g}	4	4.548	0.969	Shape
			5	5.956	0.166	Feshbach
			8	7.373	0.092	Feshbach
			11	8.703	0.241	Feshbach
			12	9.953	1.023	Shape
$^2\Sigma_u^-$	A_u	B_{1u}	3	3.097	0.737	Shape
			4	7.190	0.717	Shape

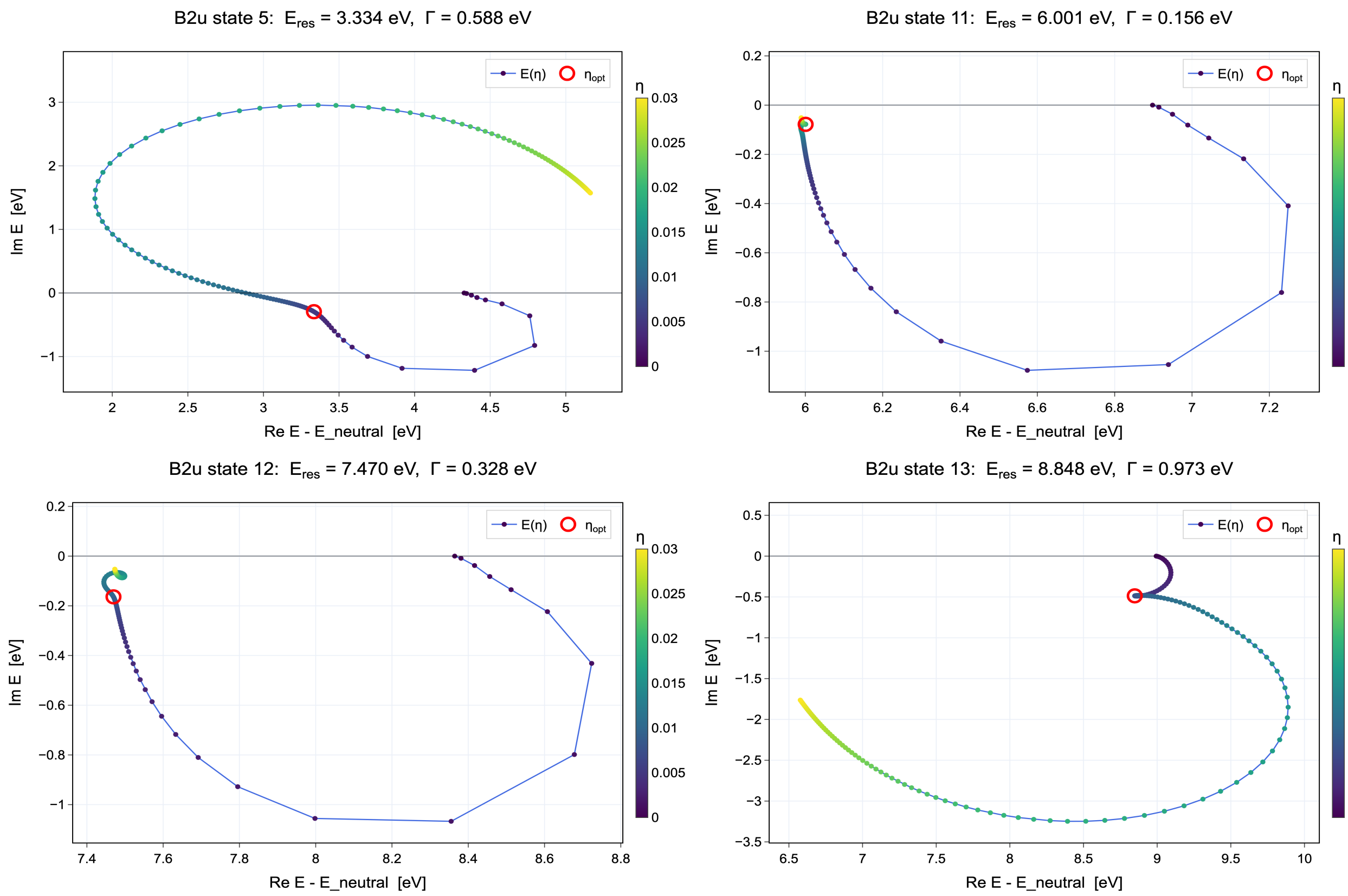

Fig S5.1. CAP η-trajectories for the B_{2u} EOM-EA transitions obtained by electron attachment to the excited-state of O_2 , $a^1\Delta_g$. The x-axis represents the real part of the resonance energy ($Re\ E$) relative to the neutral ground state, while the y-axis shows the imaginary part ($Im\ E$) The red circle denotes the optimal CAP strength (η_{opt}) determined from the trajectory analysis. The resonance energy (E_{res}) and decay width (Γ) evaluated at (η_{opt}) are indicated in the title of each panel.

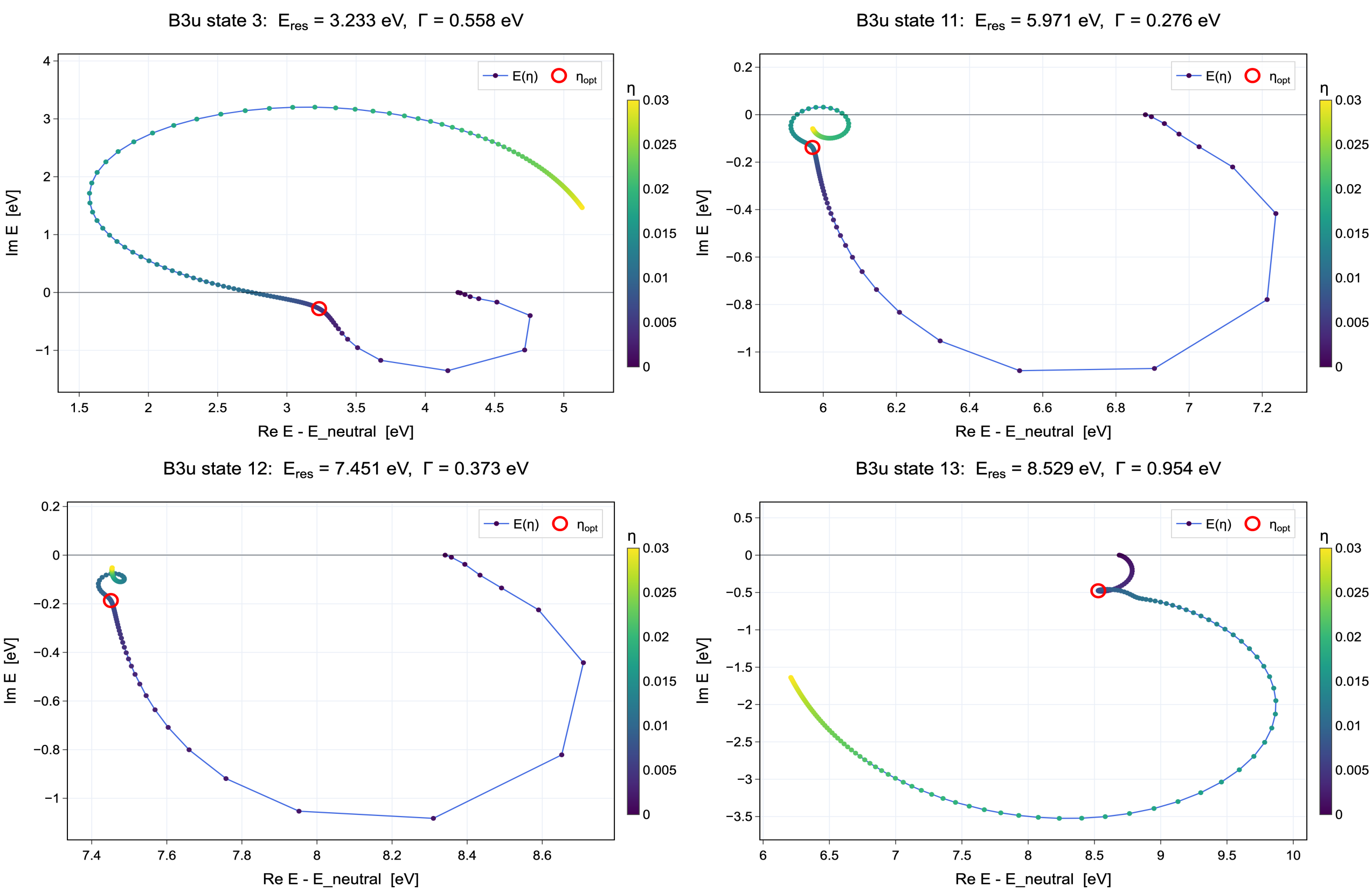

Fig S5.2. CAP η-trajectories for the B_{3u} EOM-EA transitions obtained by electron attachment to the excited-state of O_2 , $a^1\Delta_g$. The x-axis represents the real part of the resonance energy ($Re\ E$) relative to the neutral ground state, while the y-axis shows the imaginary part ($Im\ E$) The red circle denotes the optimal CAP strength (η_{opt}) determined from the trajectory analysis. The resonance energy (E_{res}) and decay width (Γ) evaluated at (η_{opt}) are indicated in the title of each panel.

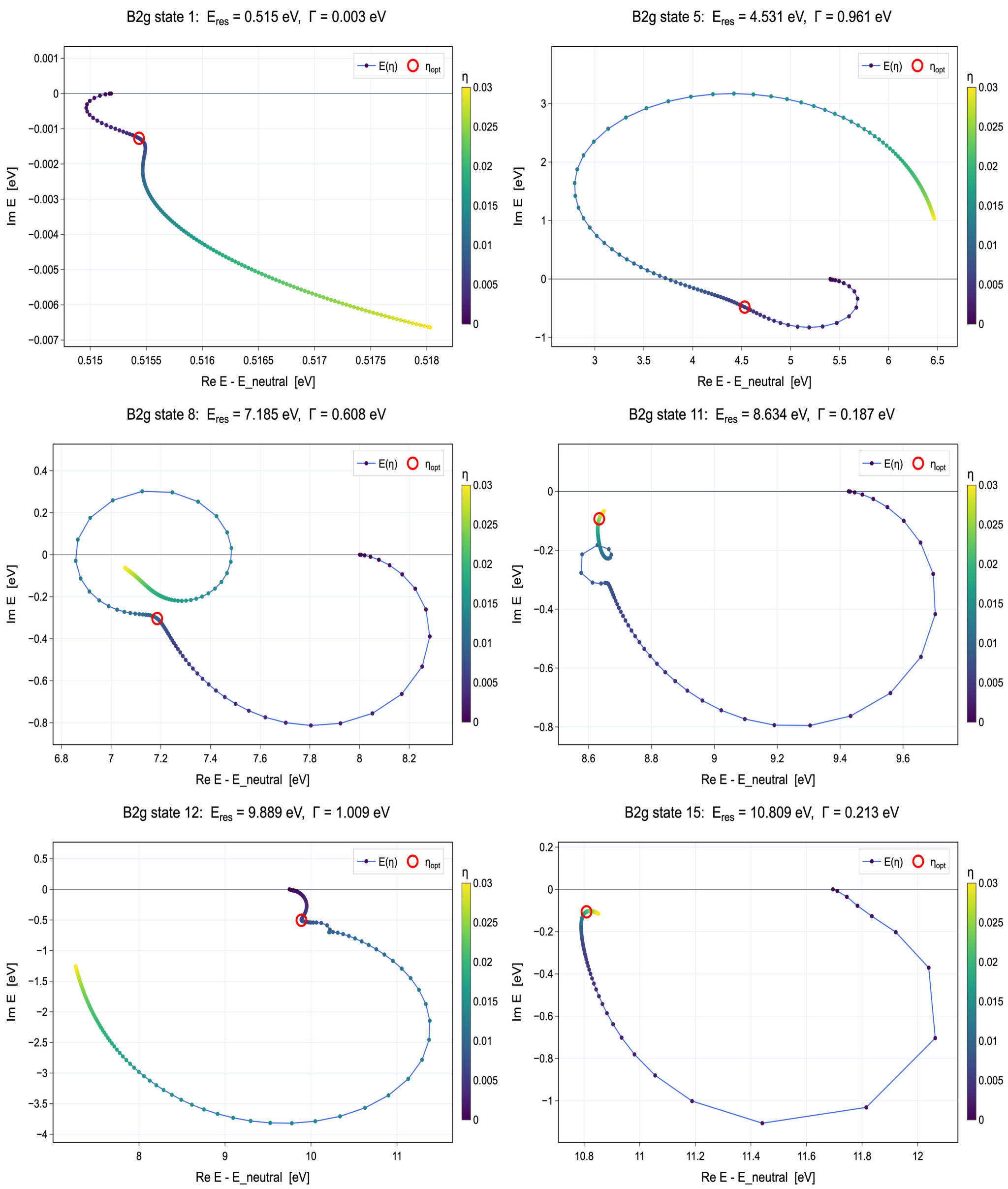

Fig S5.3. CAP η-trajectories for the B_{2g} EOM-EA transitions obtained by electron attachment to the excited-state of O_2 , $a^1\Delta_g$. The x-axis represents the real part of the resonance energy ($Re\ E$) relative to the neutral ground state, while the y-axis shows the imaginary part ($Im\ E$) The red circle denotes the optimal CAP strength (η_{opt}) determined from the trajectory analysis. The resonance energy (E_{res}) and decay width (Γ) evaluated at (η_{opt}) are indicated in the title of each panel.

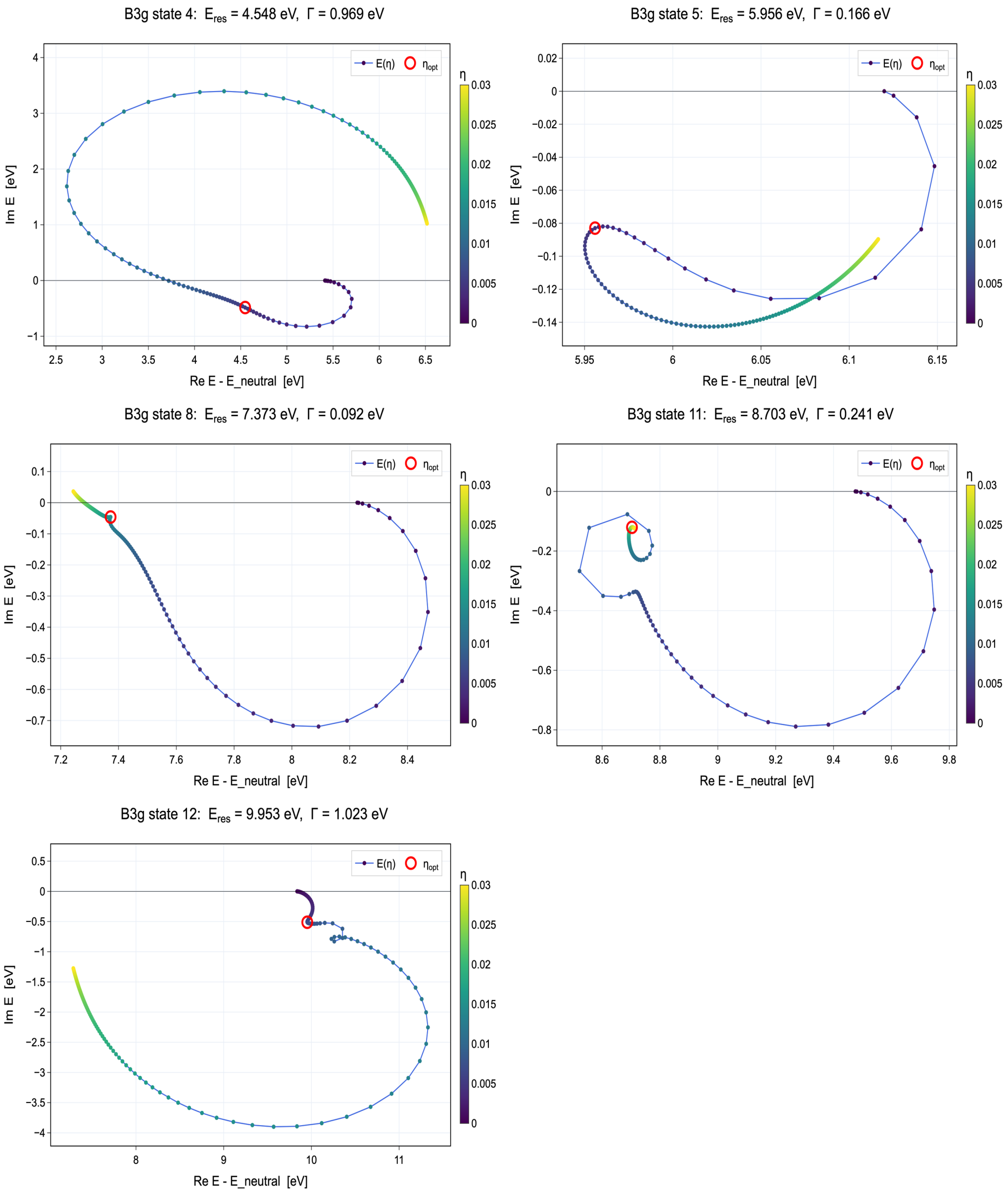

Fig S5.4. CAP η-trajectories for the B_{3g} EOM-EA transitions obtained by electron attachment to the excited-state of O_2 , $a^1\Delta_g$. The x-axis represents the real part of the resonance energy ($Re\ E$) relative to the neutral ground state, while the y-axis shows the imaginary part ($Im\ E$) The red circle denotes the optimal CAP strength (η_{opt}) determined from the trajectory analysis. The resonance energy (E_{res}) and decay width (Γ) evaluated at (η_{opt}) are indicated in the title of each panel.

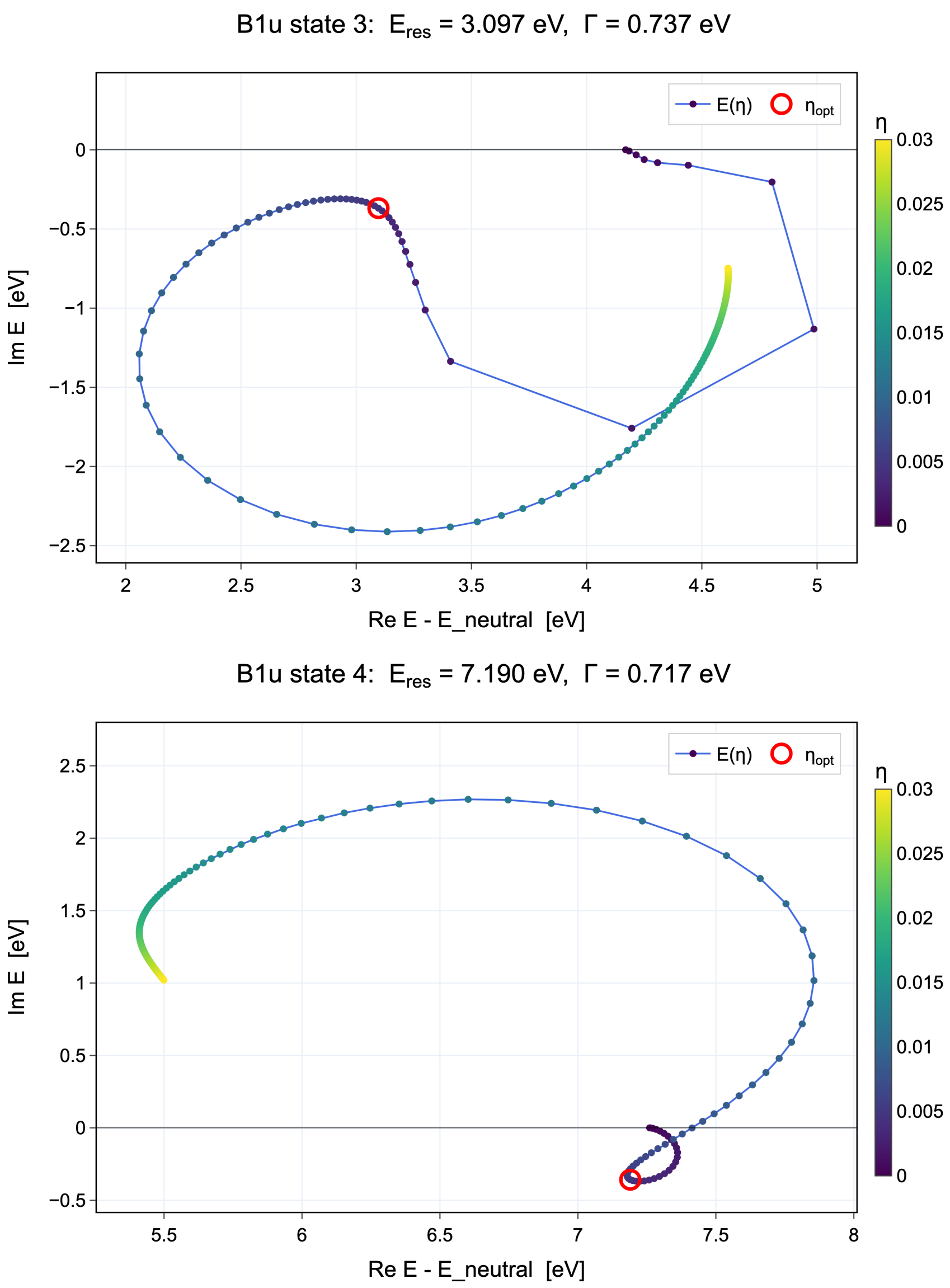

Fig S5.5. CAP η-trajectories for the B_{1u} EOM-EA transitions obtained by electron attachment to the excited-state of O_2 , $a^1\Delta_g$. The x-axis represents the real part of the resonance energy ($Re\ E$) relative to the neutral ground state, while the y-axis shows the imaginary part ($Im\ E$) The red circle denotes the optimal CAP strength (η_{opt}) determined from the trajectory analysis. The resonance energy (E_{res}) and decay width (Γ) evaluated at (η_{opt}) are indicated in the title of each panel.

Section S6. Resonances from Excited State ($b^1\Sigma_g^+ = Ag$)

α and β electron attachment are equivalent

Table S6.1. EOMEA resonances from the Excited State ($X^3\Sigma g^- = B1g$)

Resonances ($D_{\infty h}$)	D_{2h}	EOMEA Transition	State No.	Resonance Position (E_r) {eV}	Γ {eV}	Type
$^2\Pi_u$	$B_{2u} + B_{3u}$	B_{2u}	**7**	**6.251**	**0.049**	**Feshbach**
			10	7.781	0.055	Feshbach
		B_{3u}	3	2.350	0.559	Shape
			**7**	**6.270**	**0.028**	**Feshbach**
			8	5.861	0.238	Feshbach
			10	7.729	0.921	Shape
$^2\Pi_g$	$B_{2g} + B_{3g}$	B_{2g}	2	3.584	0.951	Shape
			**4**	**6.241**	**0.278**	**Feshbach**
			6	7.202	0.068	Feshabch
			8	8.921	1.026	Shape
		B_{3g}	1	0.462	0.006	Shape
			3	3.649	0.939	Shape
			6	7.040	0.647	Feshbach
			8	8.995	0.819	Shape
			10	8.876	0.997	Feshbach
$^2\Sigma_u^-$	A_u	B_{1u}	3	2.176	0.722	Shape
			**6**	**6.291**	**0.736**	**Shape**
			7	5.555	0.788	Feshbach
			9	9.886	0.738	Feshbach

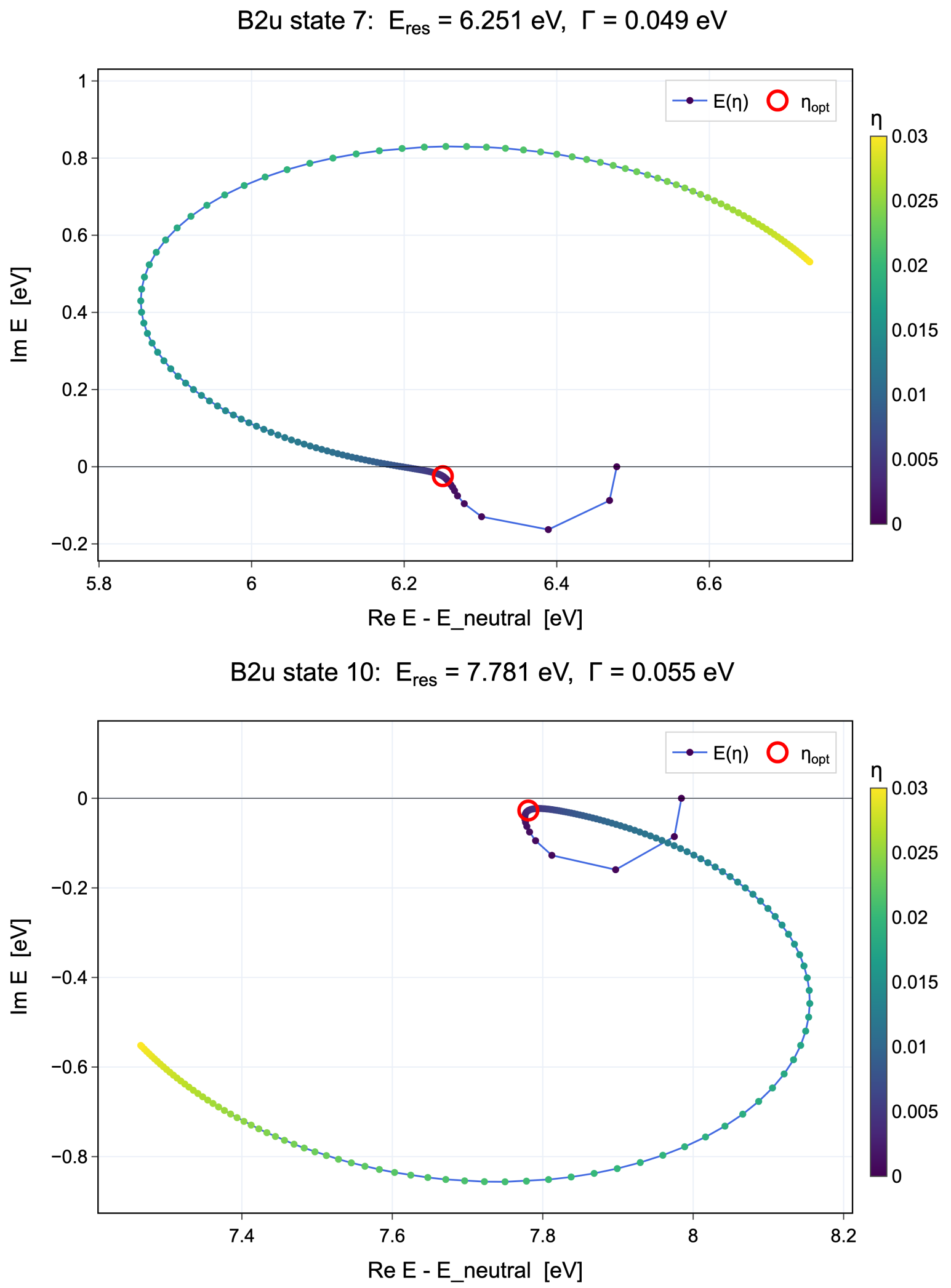

Fig S6.1. CAP η-trajectories for the B_{2u} EOM-EA transitions obtained by electron attachment to the excited-state of O_2 , $b^1\Sigma_g^+$. The x-axis represents the real part of the resonance energy ($Re\ E$) relative to the neutral ground state, while the y-axis shows the imaginary part ($Im\ E$) The red circle denotes the optimal CAP strength (η_{opt}) determined from the trajectory analysis. The resonance energy (E_{res}) and decay width (Γ) evaluated at (η_{opt}) are indicated in the title of each panel.

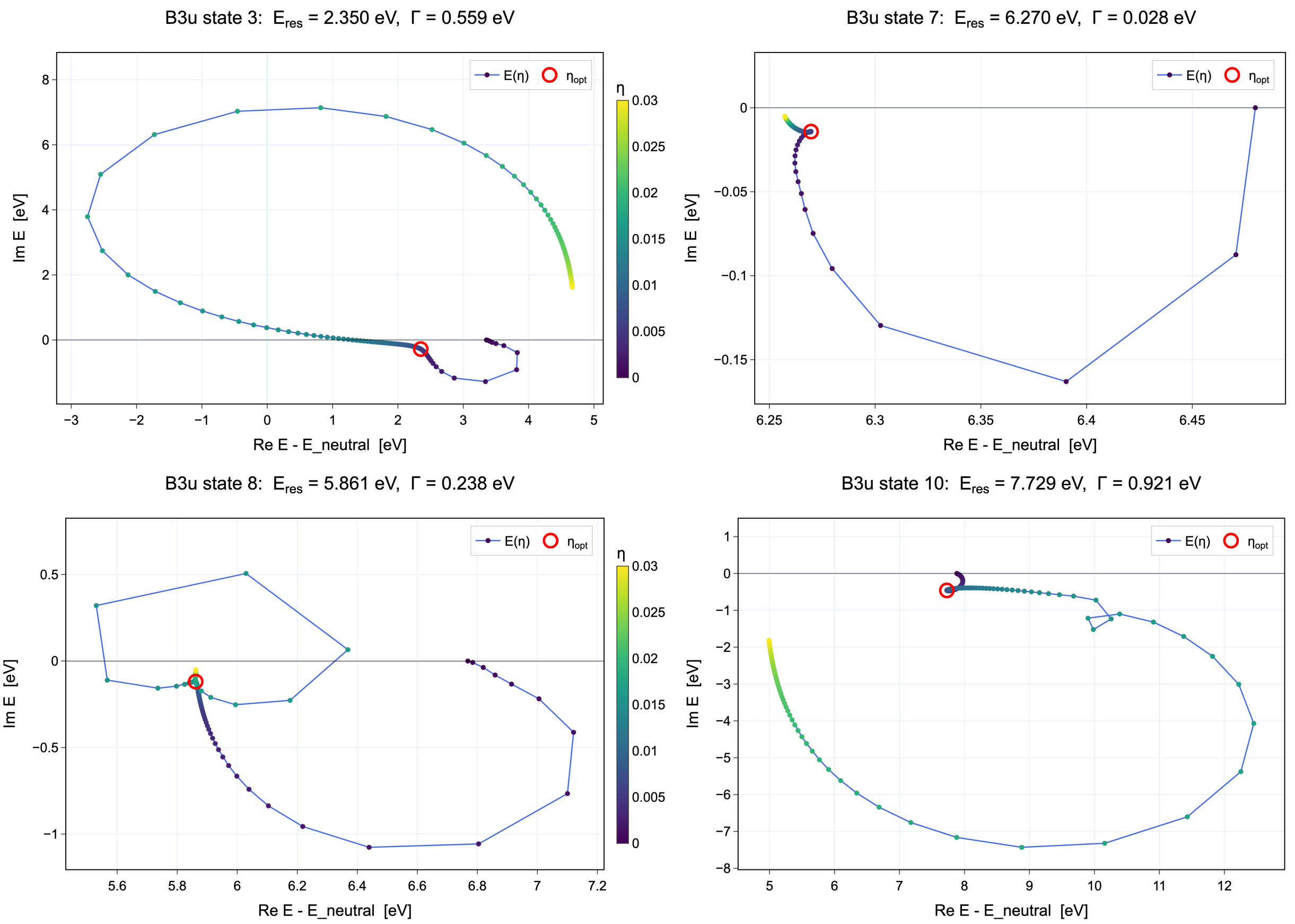

Fig S6.2 CAP η-trajectories for the B_{3u} EOM-EA transitions obtained by electron attachment to the excited-state of O_2 , $b^1\Sigma_g^+$. The x-axis represents the real part of the resonance energy ($Re\ E$) relative to the neutral ground state, while the y-axis shows the imaginary part ($Im\ E$) The red circle denotes the optimal CAP strength (η_{opt}) determined from the trajectory analysis. The resonance energy (E_{res}) and decay width (Γ) evaluated at (η_{opt}) are indicated in the title of each panel.

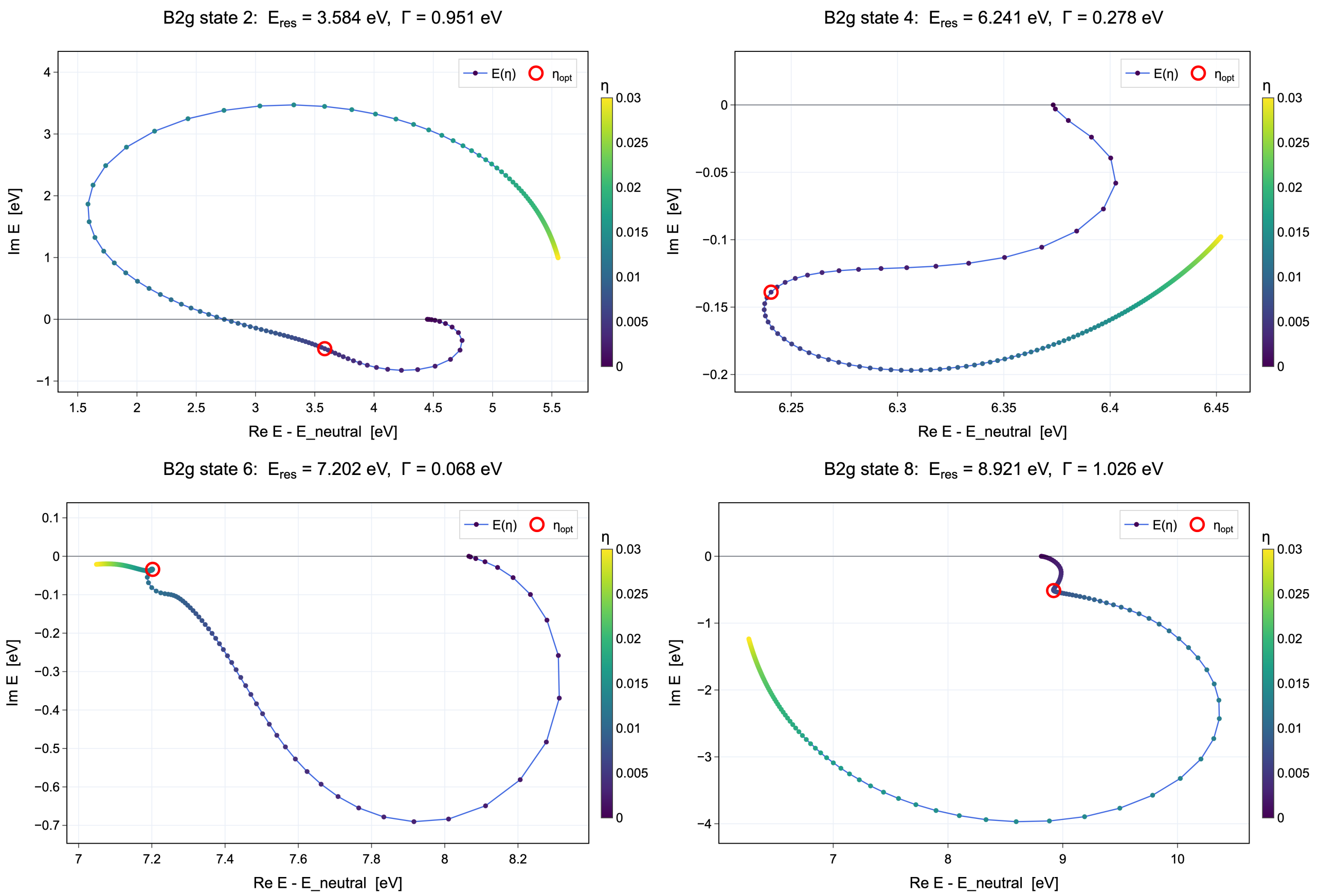

Fig S6.3 CAP η-trajectories for the B_{2g} EOM-EA transitions obtained by electron attachment to the excited-state of O_2 , $b^1\Sigma_g^+$. The x-axis represents the real part of the resonance energy ($Re\ E$) relative to the neutral ground state, while the y-axis shows the imaginary part ($Im\ E$) The red circle denotes the optimal CAP strength (η_{opt}) determined from the trajectory analysis. The resonance energy (E_{res}) and decay width (Γ) evaluated at (η_{opt}) are indicated in the title of each panel.

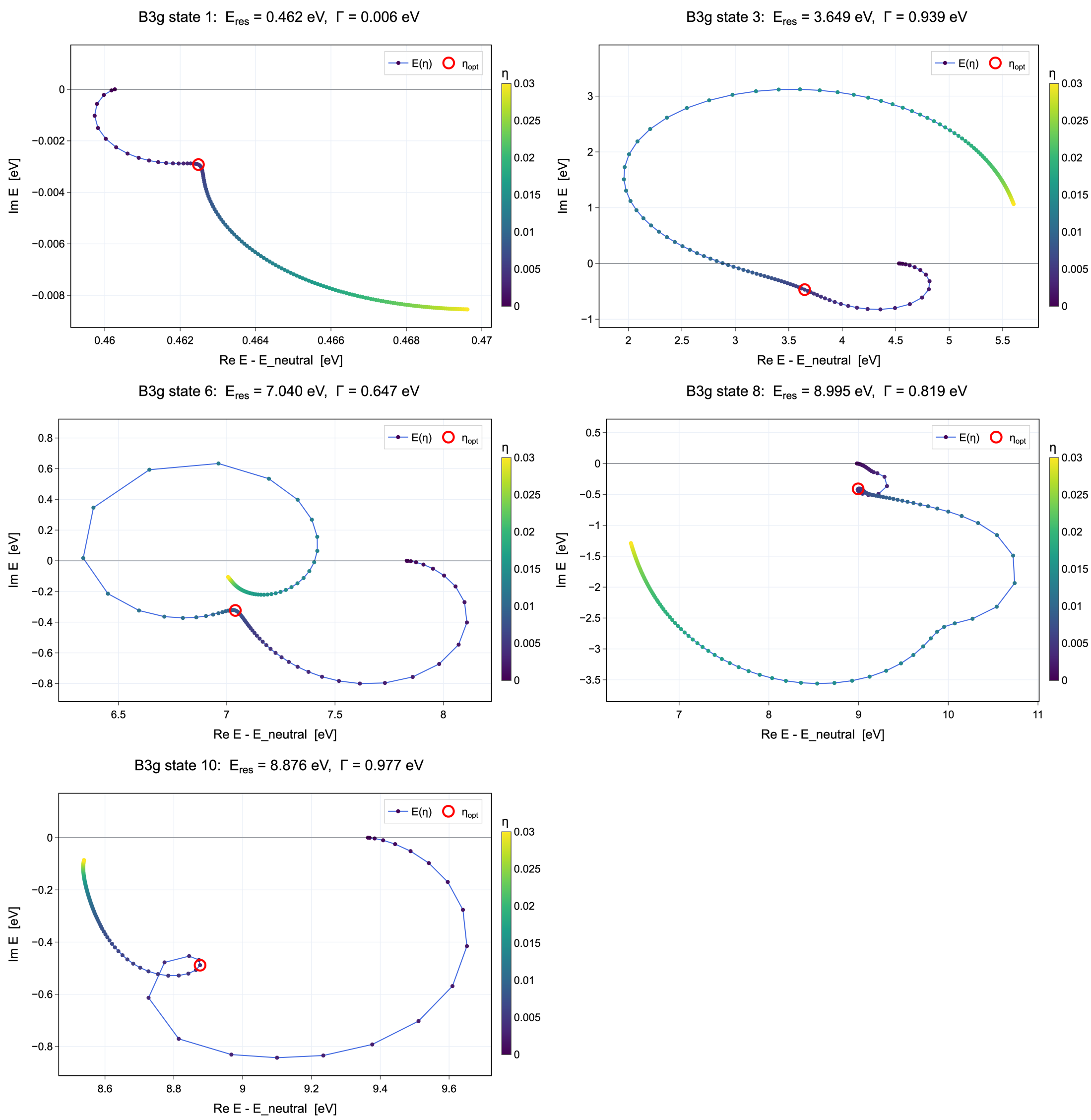

Fig S6.4. CAP η-trajectories for the B_{3g} EOM-EA transitions obtained by electron attachment to the excited-state of O_2, $b^1\Sigma_g^+$. The x-axis represents the real part of the resonance energy ($Re\ E$) relative to the neutral ground state, while the y-axis shows the imaginary part ($Im\ E$) The red circle denotes the optimal CAP strength (η_{opt}) determined from the trajectory analysis. The resonance energy (E_{res}) and decay width (Γ) evaluated at (η_{opt}) are indicated in the title of each panel.

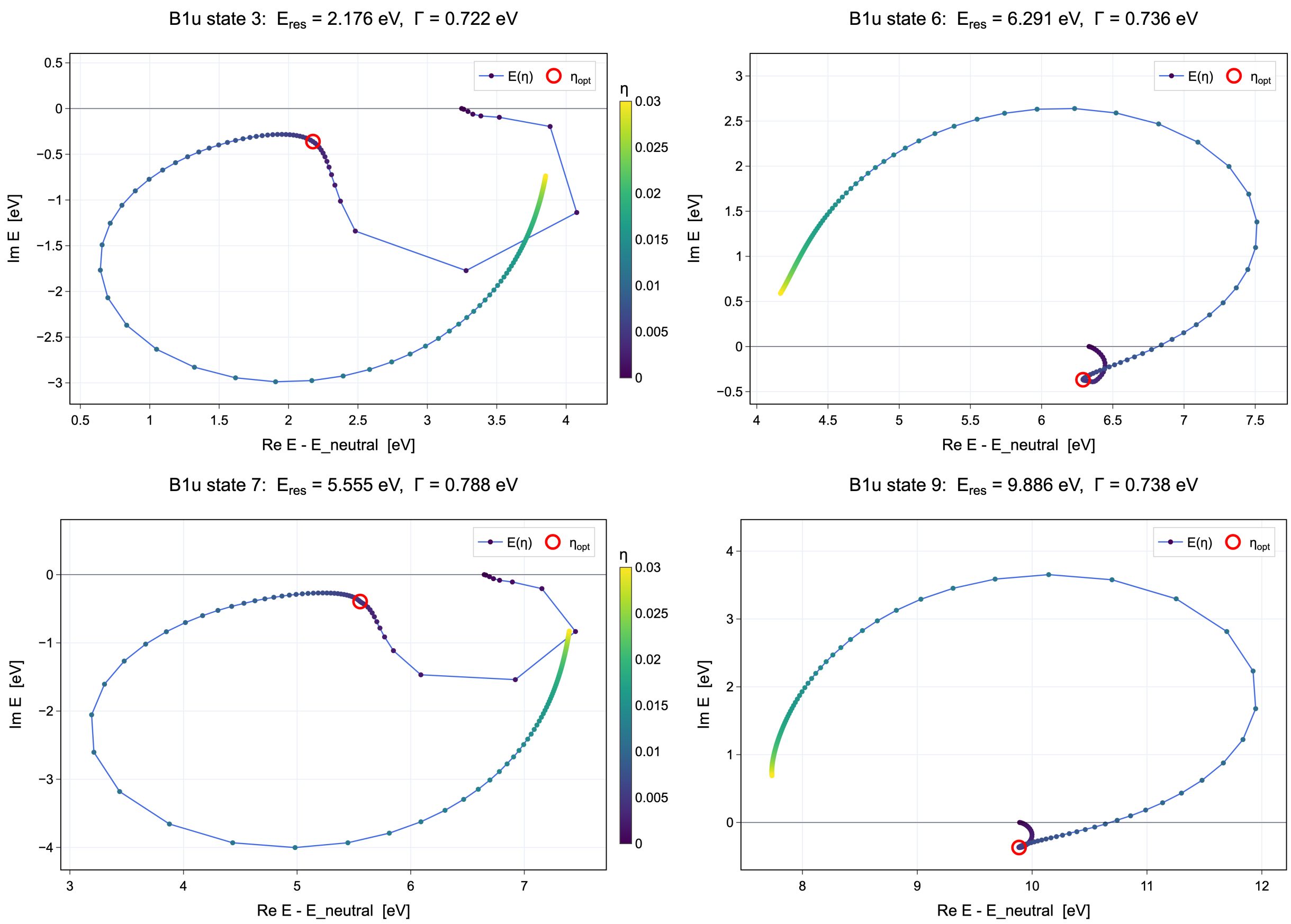

Fig S6.5. CAP η-trajectories for the B_{1u} EOM-EA transitions obtained by electron attachment to the excited-state of O_2 , $b^1\Sigma_g^+$. The x-axis represents the real part of the resonance energy ($Re\ E$) relative to the neutral ground state, while the y-axis shows the imaginary part ($Im\ E$) The red circle denotes the optimal CAP strength (η_{opt}) determined from the trajectory analysis. The resonance energy (E_{res}) and decay width (Γ) evaluated at (η_{opt}) are indicated in the title of each panel.

Section S7. Dyson-Orbital-Based O'Malley–Taylor Decomposition for Angular Distributions

The angular distribution of O^- fragments from dissociative electron attachment (DEA),

$$e^- + O_2(X\,^3\Sigma_g^-) \;\rightarrow\; O_2^{-*} \;\rightarrow\; O^- + O \tag{1}$$

is governed by the entrance-channel electron capture step under the axial-recoil approximation. Because dissociation is fast compared to molecular rotation, the O^- fragment recoils along the internuclear axis, and the laboratory-frame scattering angle θ with respect to the electron beam coincides with the molecular-frame polar angle of the captured electron's momentum. The angular distribution is then determined entirely by the capture amplitude and can be written in the partial-wave form given by O'Malley and Taylor:

$$I(\theta) = \sum_{\mu=\pm\Lambda} \left| \sum_{l} a_{l,\mu}\, e^{i\delta_l}\, Y_{l,\mu}(\theta, 0) \right|^2 \tag{2}$$

where Λ is the projection of the resonance electronic angular momentum along the molecular axis ($\Lambda = 0$ for Σ, 1 for Π, 2 for Δ states), $a_{l,\mu}$ are the partial-wave capture amplitudes, δ_l are the partial-wave scattering phase shifts, and $Y_{l,\mu}$ are the spherical harmonics. The selection rule

$$\mu = \Lambda_{\text{res}} - \Lambda_{\text{target}} = \Lambda_{\text{res}} \tag{3}$$

follows from the Σ symmetry ($\Lambda_{\text{target}} = 0$) of the O_2 ground state. The gerade/ungerade symmetry of the resonance further restricts the sum to even-l (g) or odd-l (u) terms only, which guarantees the forward–backward symmetry $I(\theta) = I(\pi - \theta)$ required for a homonuclear target.

The partial-wave coefficients $a_{l,\mu}$ are obtained from the Dyson orbital $\phi_d(\mathbf{r})$ of each resonance state, computed here at the (CAP-)EOM-EA-CCSD level. The capture amplitude in the molecular frame is defined as

$$\mathcal{M}(\hat{\mathbf{k}}) = [\tilde{\phi}_d(\mathbf{k})]^*\big|_{|\mathbf{k}|=k_0} \tag{4}$$

where $\tilde{\phi}_d(\mathbf{k})$ is the three-dimensional Fourier transform of the Dyson orbital evaluated on the momentum-space sphere of radius $k_0 = \sqrt{2\,E_{\text{inc}}}$ (atomic units), with E_{inc} the incident electron energy at resonance. The complex conjugation enforces the convention of an incoming plane wave $e^{+i\mathbf{k}\cdot\mathbf{r}}$.

The partial-wave amplitudes are then obtained by angular projection:

$$a_{l,\mu} = \int Y_{l,\mu}^*(\hat{\mathbf{k}})\; \mathcal{M}(\hat{\mathbf{k}})\; d\Omega_{\mathbf{k}} \tag{5}$$

The Dyson orbital is read from a Gaussian cube file, zero-padded to four times its original grid dimensions in each Cartesian direction, and Fourier-transformed via a three-dimensional fast Fourier

transform (FFT). For complex-valued CAP-EOM-EA orbitals, the real and imaginary parts are read from separate cube files and combined before transformation. A phase correction

$$\tilde{\phi}_d(\mathbf{k}) \;\rightarrow\; \tilde{\phi}_d(\mathbf{k})\, e^{i\mathbf{k}\cdot\mathbf{R}} \tag{6}$$

is applied to shift the coordinate origin to the bond midpoint **R**, which is essential for clean gerade/ungerade separation in homonuclear systems.

The transformed orbital is interpolated onto the k_0-sphere using third-order spline interpolation, with sampling points defined in the molecular frame (the z-axis aligned along the O–O bond). The angular integrals in Eq. (4) are evaluated numerically using Gauss–Legendre quadrature in θ and uniform quadrature in ϕ, with the number of quadrature points chosen to ensure convergence up to $l_{\max}$.

After projection, symmetry is enforced by retaining only the components with $\mu = \pm\Lambda$ and the correct l-parity (even for gerade, odd for ungerade resonances). The fraction of spectral weight in the discarded, symmetry-forbidden channels,

$$\varepsilon_{\text{contam}} = 1 - \frac{\sum_{(l,\mu)\in\text{allowed}} \left|a_{l,\mu}\right|^2}{\sum_{l,\mu} \left|a_{l,\mu}\right|^2} \tag{7}$$

serves as a diagnostic of grid quality and orbital alignment, and is typically below 1–2%.

The magnitudes $\left|a_{l,\mu}\right|$ and geometric phases $(-i)^l$ are fully determined by the Dyson orbital. The scattering phase shifts δ_l, however, are continuum quantities not contained in a bound-state or resonance orbital. In the limit where a single partial wave dominates, channel carries the dominant fraction of the capture probability the angular distribution is independent of δ_l and is entirely predicted by the orbital. When multiple partial waves contribute comparably, the δ_l must be supplied from separate scattering calculations or treated as parameters (here from experimental data); they control the interference pattern (lobe positions) but not the overall shape symmetry, which is fixed by Λ and the g/u parity.

When several resonance states contribute at overlapping energies, the total angular distribution is obtained as the incoherent (intensity-weighted) sum

$$I_{\text{total}}(\theta) = \sum_i w_i\, I_i(\theta), \tag{8}$$

where w_i reflects the relative cross section or branching ratio of each resonance channel i.

For our calculation we have taken the dyson orbital from CAP-EOM-EA-CCSD calculation.
$Re(\phi^{lr})$ orbital was taken for each resonance near ~6.5 eV. The scattering phase shifts δ_l were taken from experimental data.

Dyson Orbitals of Resonances and Angular Distribution

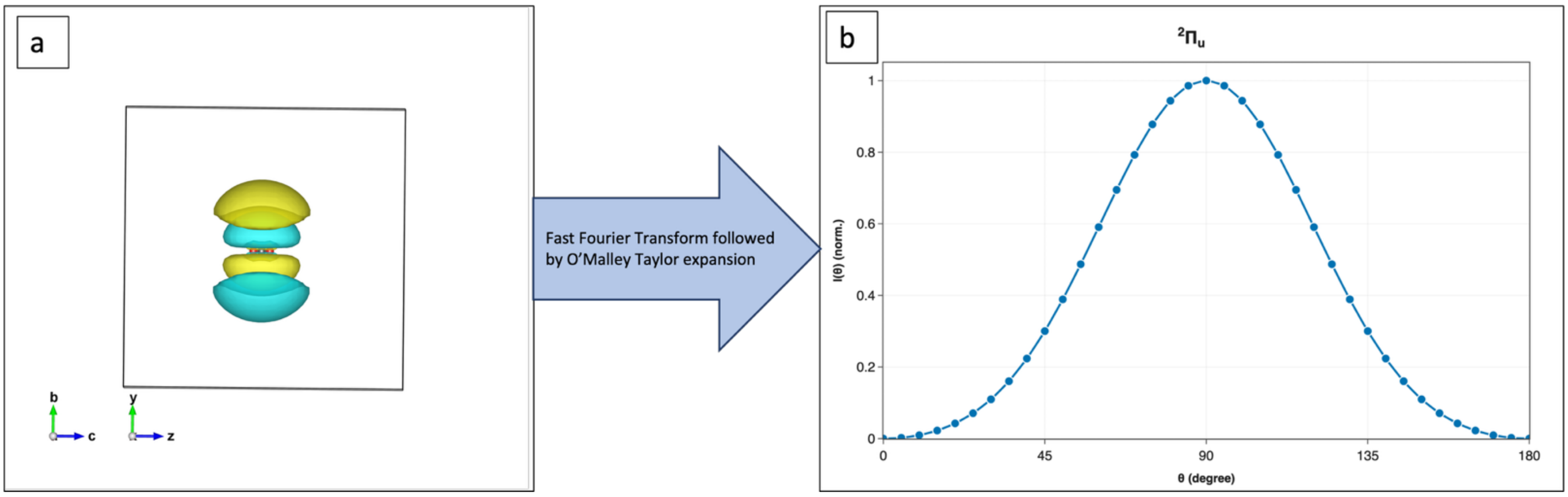

Figure S7.1. (a) Dyson orbital corresponding to the $^2\Pi_u$ resonance state. (b) Angular distribution of O^- fragments obtained from the Dyson orbital by applying a Fast Fourier Transform (FFT) to generate the momentum-space wavefunction, followed by an O'Malley–Taylor partial-wave analysis. The distribution shown includes only the contribution from the $^2\Pi_u$ symmetry channel

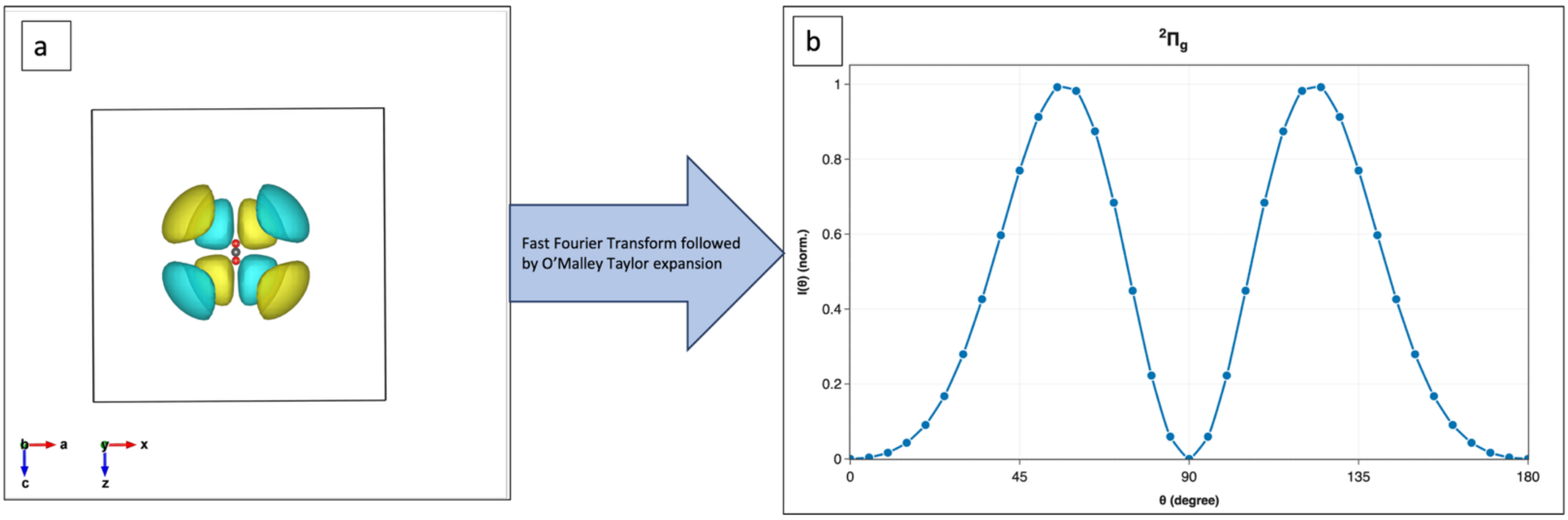

Figure S7.2. (a) Dyson orbital corresponding to the $^2\Pi_g$ resonance state. (b) Angular distribution of O^- fragments obtained from the Dyson orbital by applying a Fast Fourier Transform (FFT) to generate the momentum-space wavefunction, followed by an O'Malley–Taylor partial-wave analysis. The distribution shown includes only the contribution from the $^2\Pi_g$ symmetry channel

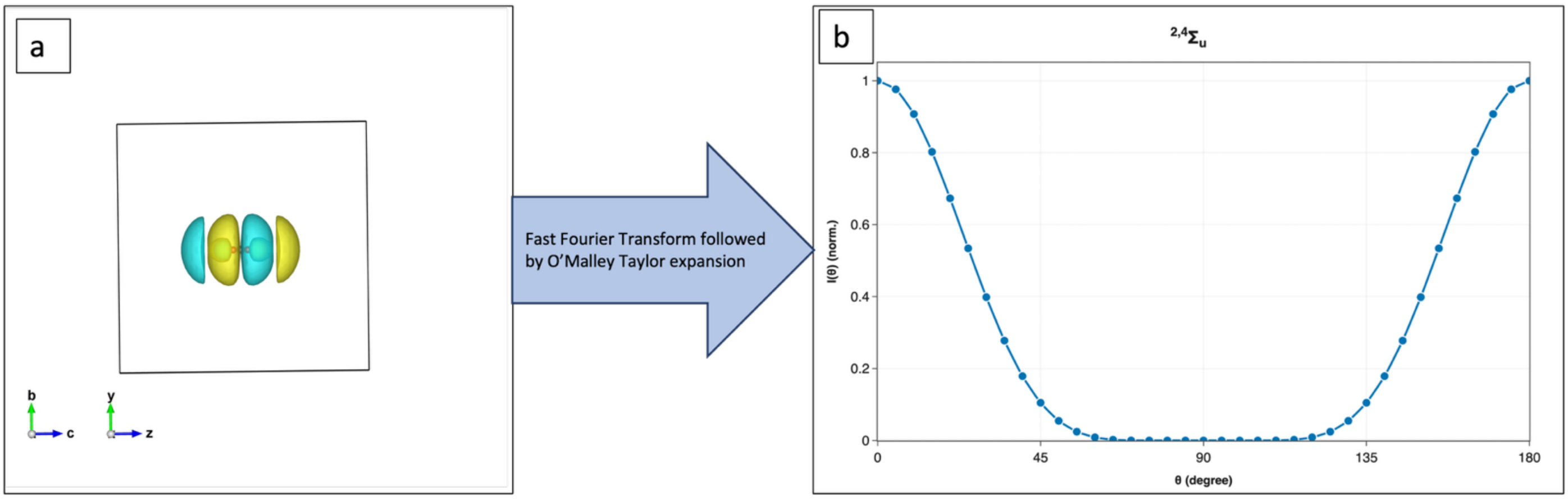

Figure S7.2. (a) Dyson orbital corresponding to the $^{2,4}\Sigma_u$ resonance state. (b) Angular distribution of O^- fragments obtained from the Dyson orbital by applying a Fast Fourier Transform (FFT) to generate the momentum-space wavefunction, followed by an O'Malley–Taylor partial-wave analysis. The distribution shown includes only the contribution from the $^{2,4}\Sigma_u$ symmetry channel

Section S8. Complete Basis Set

The CBS extrapolation was done using L3(Two point extrapolation) and EX1 (Three point extrapolation). EX1 was used where three energies were available.

Table S8.1 CBS extrapolation for O_2 Ground State and Excited State from XMS-CASPT2 calculation

State	aug-cc-pVDZ	aug-cc-pVTZ	aug-cc-pVQZ	CBS: L3 (T,Q)	CBS: EX1 (D,T,Q)
X $^3\Sigma g^-$	−150.00289821	−150.12056497	−150.16045462	−150.18956329	−150.18091290
a $^1\Delta g$	−149.96598444	−150.08556647	−150.12606891	−150.15562475	−150.14681317
b $^1\Sigma g^+$	−149.94138366	−150.06059944	−150.10116488	−150.13076670	−150.12208730

Table S8.1.1 Excitation energies relative to X $^3\Sigma g^-$

State	CBS: L3 (T,Q) {eV}	CBS: EX1 (D,T,Q) {eV}
a $^1\Delta g$	0.9235	0.9279
b $^1\Sigma g^+$	1.5999	1.6007

Table S8.2 CBS extrapolation of O_2 Ground state from CCSD calculation

X $^3\Sigma g^-$	aug-cc-pvdz+3s3p3d	aug-cc-pvtz+3s3p3d	aug-cc-pvqz+3s3p3d	CBS: L3 (T,Q)	CBS: EX1 (D,T,Q)
Energy (Ha) from CCSD calculation	-150.01437160	-150.15056862	-150.21908203	−150.26907830	−150.28843541

Table S8.2.1 EA States from Ground State (X $^3\Sigma g^-$) electron attachment

Parentheses () in the front indicates state order

	aug-cc-pvdz+3s3p3d (in eV)	aug-cc-pvtz+3s3p3d (in eV)	aug-cc-pvqz+3s3p3d (in eV)	CBS: L3 (T,Q) (in eV)	CBS: EX1 (D,T,Q) (in eV)
$^2\Pi_g$	(1) 0.6192	(1) 0.430	(1) 0.3429	0.2793	0.2710
	-	(7) 7.017	(7) 6.9863	6.9639	-
$^2\Pi_u$	-	(3) 1.880	(3) 1.7725	1.6941	-
	-	(10) 5.819	(10) 5.7456	5.6920	-
$^2\Sigma_u^-$	-	(3) 1.683	(3) 1.5285	1.4158	-
	-	(12) 9.801	(12) 9.3850	9.0814	-
$^4\Sigma_u^-$	-	(3) 1.641	(3) 1.4978	1.3933	-
	-	(9) 11.075	(8) 10.5256	10.1247	-

Table S8.2.2 EA States from a ¹Δg state electron attachment

		aug-cc-pvdz+3s3p3d (in eV)	aug-cc-pvtz+3s3p3d (in eV)	aug-cc-pvqz+3s3p3d (in eV)	CBS: L3 (T,Q) (in eV)	CBS: EX1 (D,T,Q) (in eV)
$^2\Pi_u$	B_{2u}	-	(11) 6.001	(11) 5.9186	5.8585	-
	B_{3u}	-	(11) 5.971	(11) 5.9000	5.8482	-
$^2\Pi_g$	B_{2g}	-	(8) 7.185	(8) 7.1763	7.1700	-
	B_{3g}	-	(5) 5.956	(5) 6.0992	6.2037	-
		-	(8) 7.373	(8) 7.3710	7.3695	-
$^2\Sigma_u^-$	B_{1u}	-	(4) 7.190	(4) 6.6449	6.2471	-

Table S8.2.3 EA States from b ¹Σg⁺ electron attachment

		aug-cc-pvdz+3s3p3d (in eV)	aug-cc-pvtz+3s3p3d (in eV)	aug-cc-pvqz+3s3p3d (in eV)	CBS: L3 (T,Q) (in eV)	CBS: EX1 (D,T,Q) (in eV)
$^2\Pi_u$	B_{2u}	-	(7) 6.251	(8) 5.8848	5.6176	-
	B_{3u}	-	(4) 6.270	(8) 5.9243	5.6720	-
$^2\Pi_g$	B_{2g}	-	**(4) 6.241**	**(3) 6.3832**	**6.4870**	-
	B_{3g}	-	(7) 7.040	(6) 7.0613	7.0768	-
$^2\Sigma_u^-$	B_{1u}	-	(6) 6.291	(6) 5.8686	5.5604	-
